# Supplementary material for: Mutation-induced filaments of folded proteins are inert and non-toxic in a cellular system
Source: Mol Syst Biol. 2025 Sep 15;21(10):1306–24. doi: 10.1038/s44320-025-00144-y (PMC12494878; doi:10.1038/s44320-025-00144-y)
Supplement: Supplementary file 1 — Appendix [file 44320_2025_144_MOESM1_ESM.pdf]

Appendix for

Mutation-induced filaments of folded proteins are inert and non-toxic in a cellular system

Appendix Figure S1. Cryo-TEM of 1M3U filament shows the decamer structure. 1
Appendix Figure S2. Controlling for the impact of expression on puncta/filament formation in the hybrid-formation assay (related to Fig. 2). 2
Appendix Figure S3. Coexpression of misfolded mutant subunits with wild types. 2
Appendix Figure S4. Expression of yeast chaperones, proteasome, and stress-related proteins upon expression of SA and misfolded mutants. 3
Appendix Figure S5. colocalization 'all' categories. 4
Appendix Figure S6. Mass spectrometry following immunoprecipitation. 4
Appendix Figure S7. YFP pull-down enrichment analysis. 5
Appendix Figure S8. Competition with WT-scarlet. 6
Appendix Figure S9. Proteomics analysis. 7
Appendix Figure S10. Proteomics Enrichment. 8
Appendix Table S1. Details of homomers targeted for mutations: 9
Appendix Table S2. Manual quantifications of agglomerates in dividing cells. 10
Appendix Table S3. Summary of evidence towards an agglomerate or an aggregated state for the different constructs. **10**

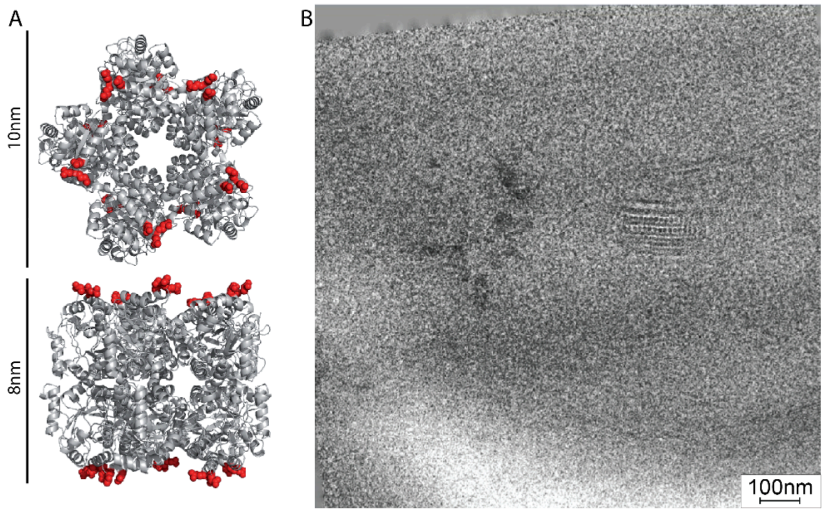

Appendix Figure S1. Cryo-TEM of 1M3U filament shows the decamer structure.

A. The X-ray structure of 1M3U. The length and height of the decamer are indicated on the side. Mutated residues are highlighted in red. **B.** Cryo-electron Tomogram of yeast cells expressing 1M3U. Individual decamers are observed.

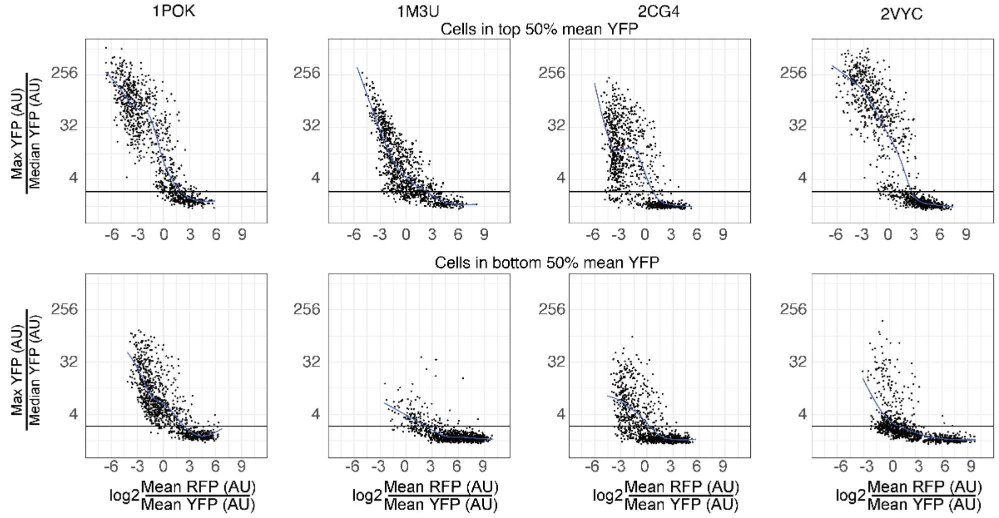

Appendix Figure S2. Controlling for the impact of expression on puncta/filament formation in the hybrid-formation assay (related to Fig. 2).

We control that wild type-to-mutant subunit ratio is more important in dictating self-assembly than the absolute abundance of mutant subunits per cell. To this aim, we consider cells with the 50% highest (top) or 50% lowest (bottom) concentration of mutant subunits. Within each group, we see that the ratio wild type-to-mutant exhibits the same effect of inhibiting self-assembly. Mutant and wild-type subunits were quantified individually in each cell based on their respective green and red fluorescence intensities. The y-axis quantifies the presence of a foci or filament in a particular cell by the ratio of maximal/median green fluorescence in that cell. Cells with a ratio above 2.5 (horizontal black line) are assigned as containing a puncta or filament. The x-axis quantifies the amount of wild-type subunit relative to the mutant in a particular cell by the ratio of red/green fluorescence.

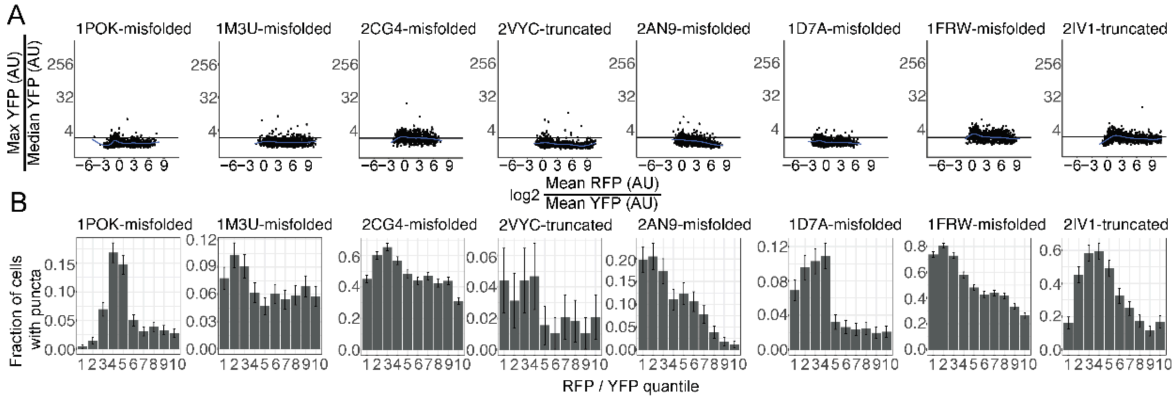

Appendix Figure S3. Coexpression of misfolded mutant subunits with wild types.

A. Quantification of a mutant's self-assembly depending on the relative expression of its corresponding wild type subunit. Misfolded- mutants were either achieved by introducing hydrophobic-to-charged mutations in the core of the protein (annotated as "misfolded"), or by truncation of the sequence (annotated as "truncated", more details are provided in Table S1). The x-axis quantifies the amount of wild-type subunit relative to the mutant in a particular cell by the ratio of red/green fluorescence. The y-axis quantifies the presence of a foci or filament in a particular cell by the ratio of maximal/median green fluorescence. Cells with a ratio above 2.5 (horizontal black line) are assigned as containing a puncta or filament. A spline fit is shown to serve as a visual guide. **B.** Same data as in panel A, where the y-axis now quantifies the fraction cells containing a foci or filament (ratio > 2.5), and the x-axis corresponds to the same ten quantiles of red/green intensity ratios. Error bars represent a 95% confidence interval.

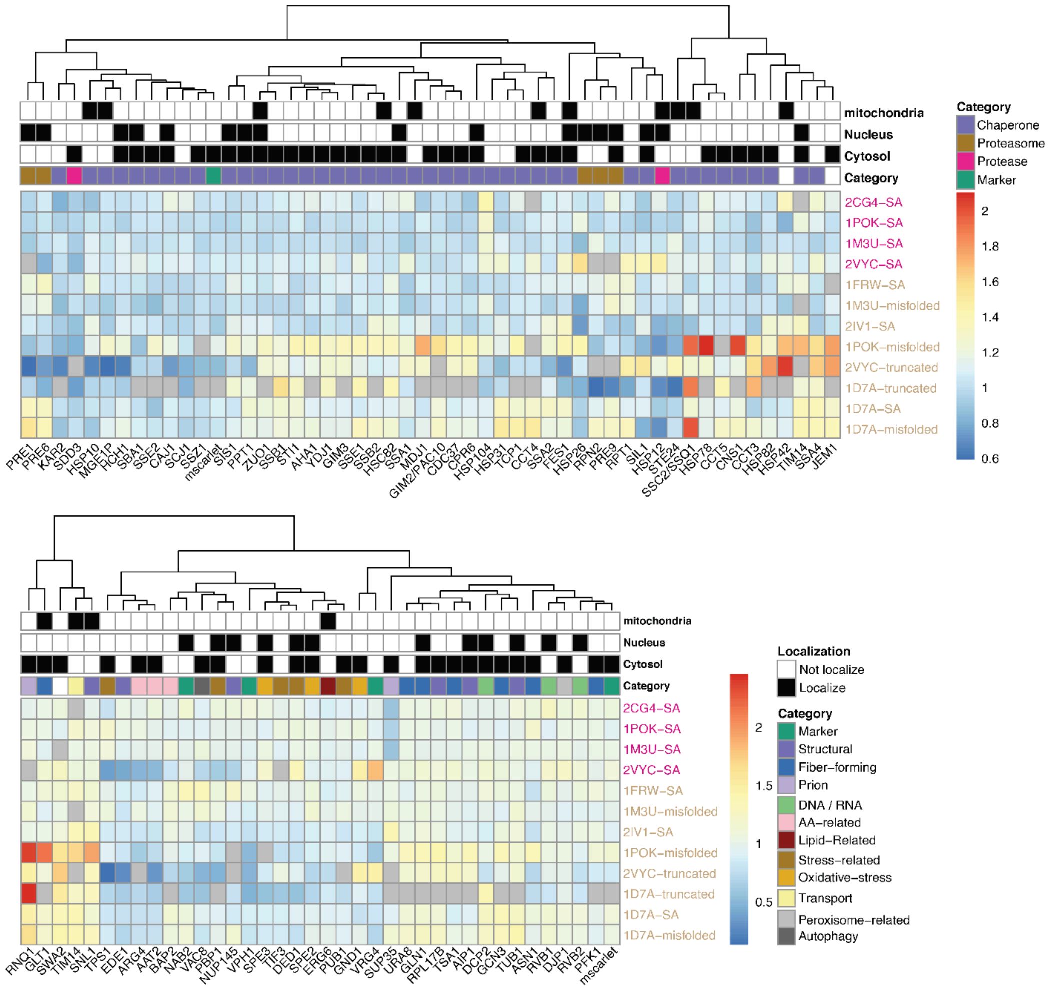

Appendix Figure S4. Expression of yeast chaperones, proteasome, and stress-related proteins upon expression of SA and misfolded mutants.

Heatmap depicting mean Scarlet expression of cellular chaperones and proteostasis proteins (top panel) or various stress and maintenance processes (bottom panel) in cells expressing either folded (magenta) or misfolded (brown) constructs. Scarlet expression is normalized to expression of cells containing wild type variants of the same construct. Only cells detected to have a foci in the YFP channel are used. NA values appear in grey. On top localization phenotype (black) and quality control type are written.

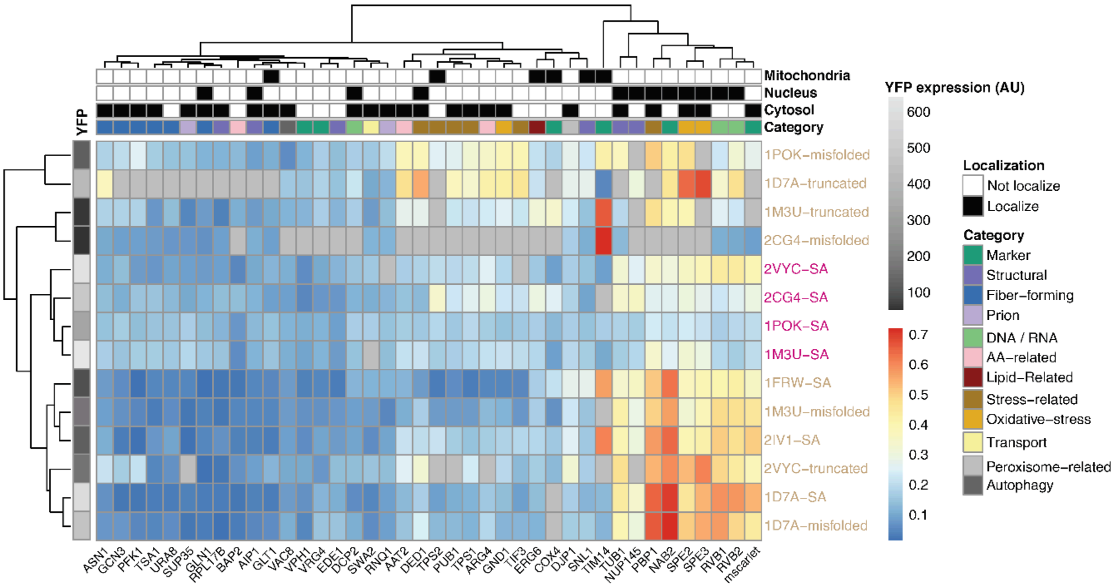

Appendix Figure S5. colocalization ‘all’ categories.

Hierarchically clustered heatmap depicting colocalization scores between various stress and maintenance processes and either folded (magenta) or misfolded (brown) constructs. Only cells detected to have at least one puncta in the YFP channel are used. NA values appear in gray and reflect an insufficient number of puncta containing cells (<20) or a lack of fluorescent signal. On top localization phenotype (black) and quality control type are written. (Left), construct mean-YFP expression (AU).

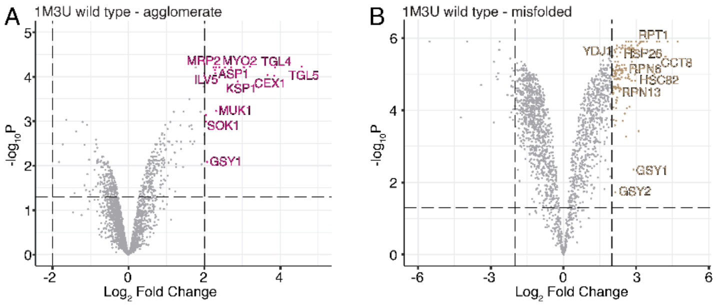

Appendix Figure S6. Mass spectrometry following immunoprecipitation.

Mass spectrometry analysis following pull-down. **A.** Volcano plots of change in protein expression between 1M3U wild type and 1M3U agglomerate mutant. Labels are some of the significant hits. **B.** Volcano plots of change in protein expression between 1M3U wild type and 1M3U misfolded mutant. Labels are some of the significant hits.

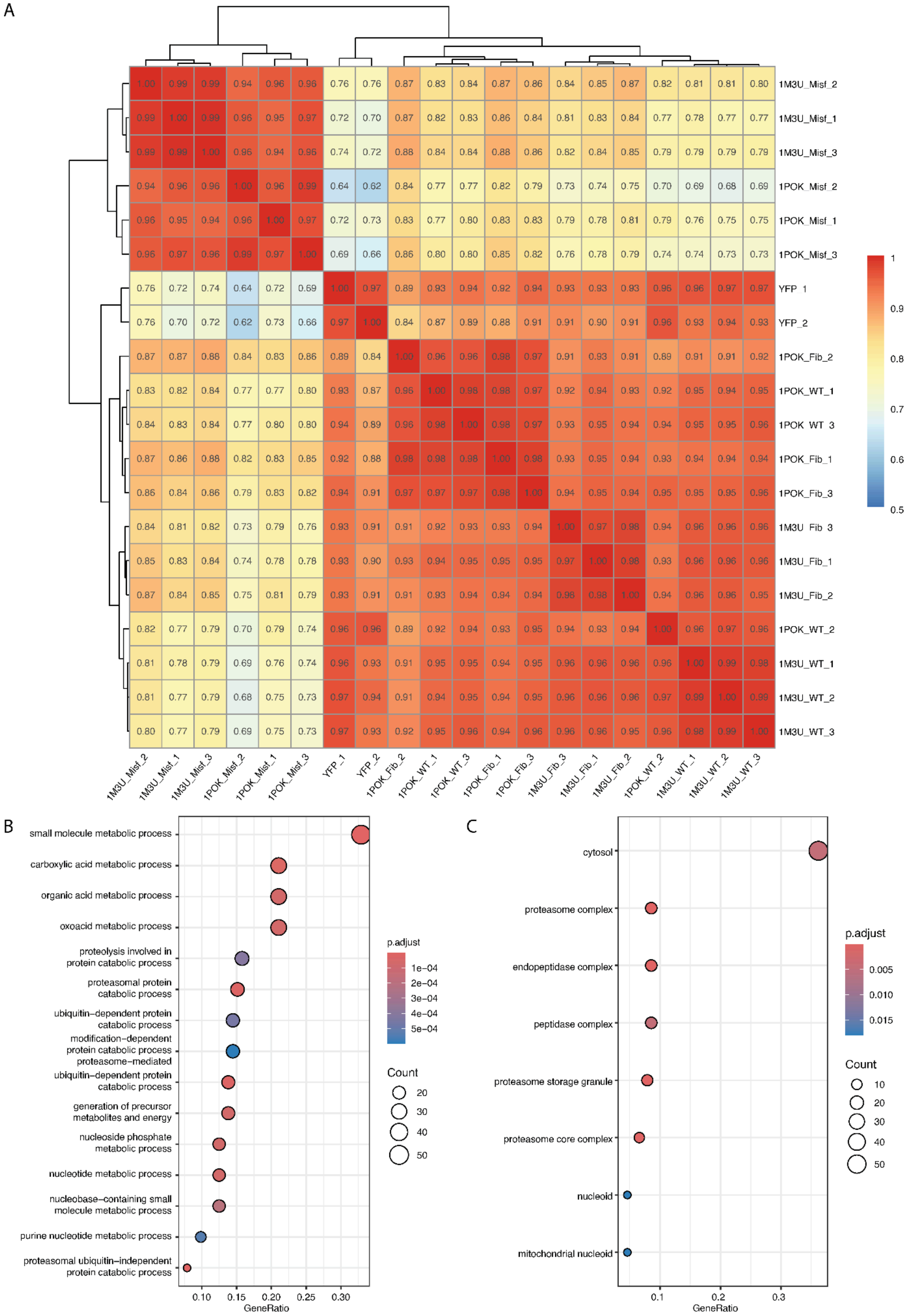

Appendix Figure S7. YFP pull-down enrichment analysis.

A. Correlogram of Mass spectrometry samples of the YFP pull-down enriched fractions **B.** GO enrichment analysis for Biological Processes (BP) among proteins over-represented in the pull-down fraction of 1POK-misfolded compared to 1POK-wild type. **C**. Same as B for Cellular Compartments (CC).

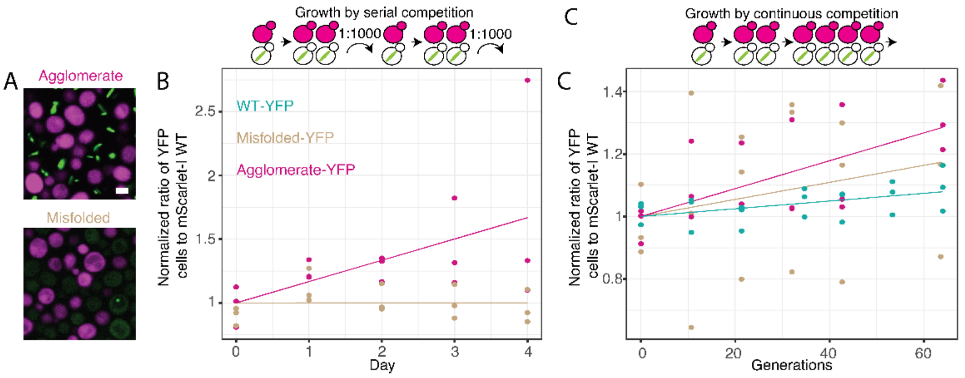

Appendix Figure S8. Competition with WT-scarlet.

A. Representative micrographs of the competing strains. Scale bar = 3µm. **B.** Serial dilution competition assay between mat-a yeast expressing scarlet fused 1POK (magenta) and mat-a transformed with Agglomerate, or Misfolded YFP fused 1POK variants (green). Competitions occurred in liquid culture and were diluted 1000-fold daily. Every day, a sample was imaged and analyzed to quantify the populations of cells from each strain. **C.** Continuous competition that occurred over 144 hours in a chemostat.

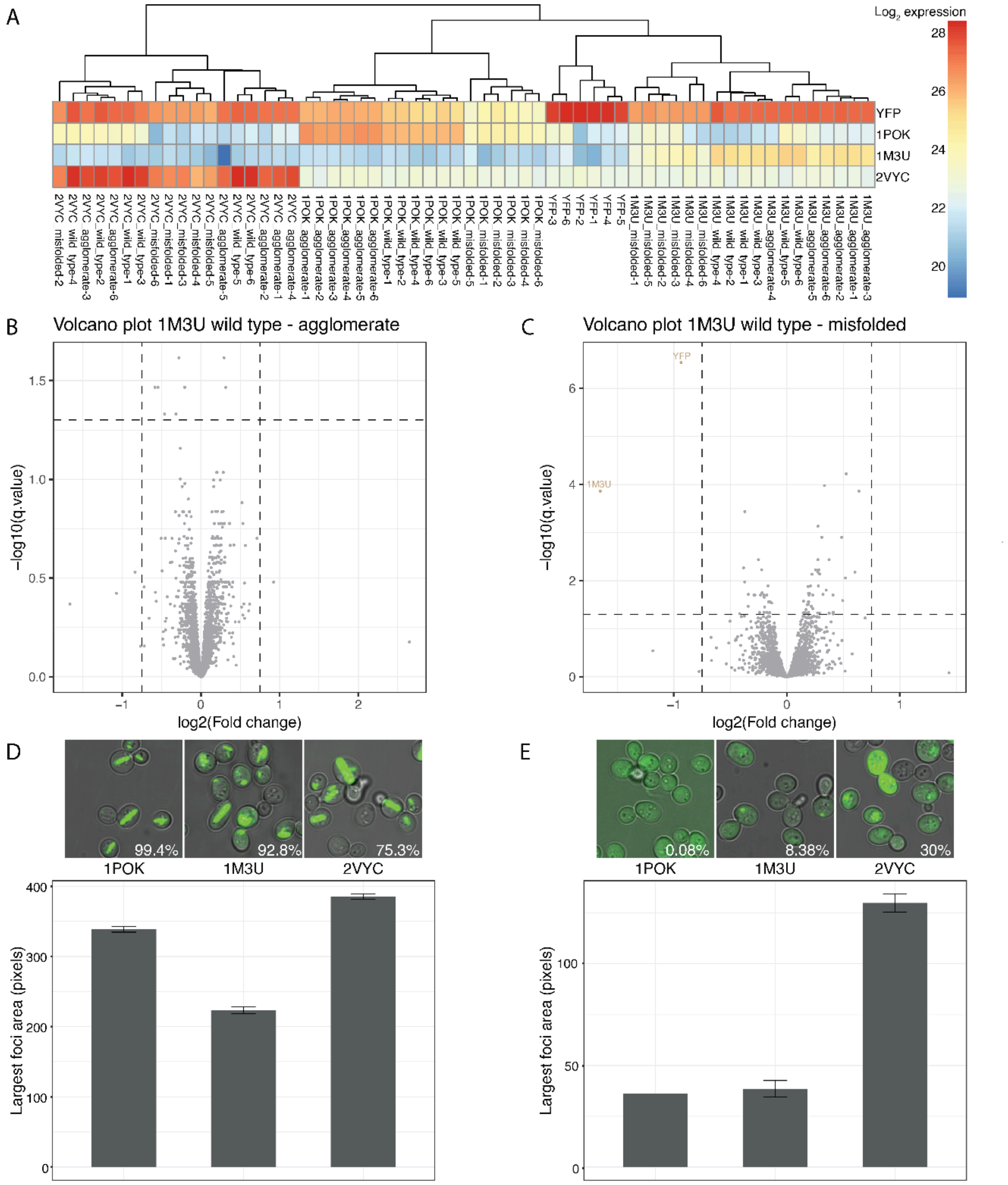

Appendix Figure S9. Proteomics analysis.

A. Heatmap of proteomics data depicting the expression of the exogenous constructs in all samples. **B-C**. Volcano plots of protein abundance differences between wild type and agglomerating (panels B) or misfolded (panel C) mutants. Each point is an average of six replicates. Pellets were frozen from a log phase liquid culture. Proteins were considered as hits if their expression changed more than 1.68 fold and had an adjusted P value < 0.05. **B.** 1M3U wild type to agglomerate. **C.** 1M3U wild type to misfolded. **D-E.** (top) representative micrographs of strain used in microscopy. Percentage of cells with foci written in white. (bottom) Quantification of the area of the largest foci in cells that contain a foci. **D.** Constructs expressing agglomerate mutants. **E.** Constructs expressing misfolded mutants.

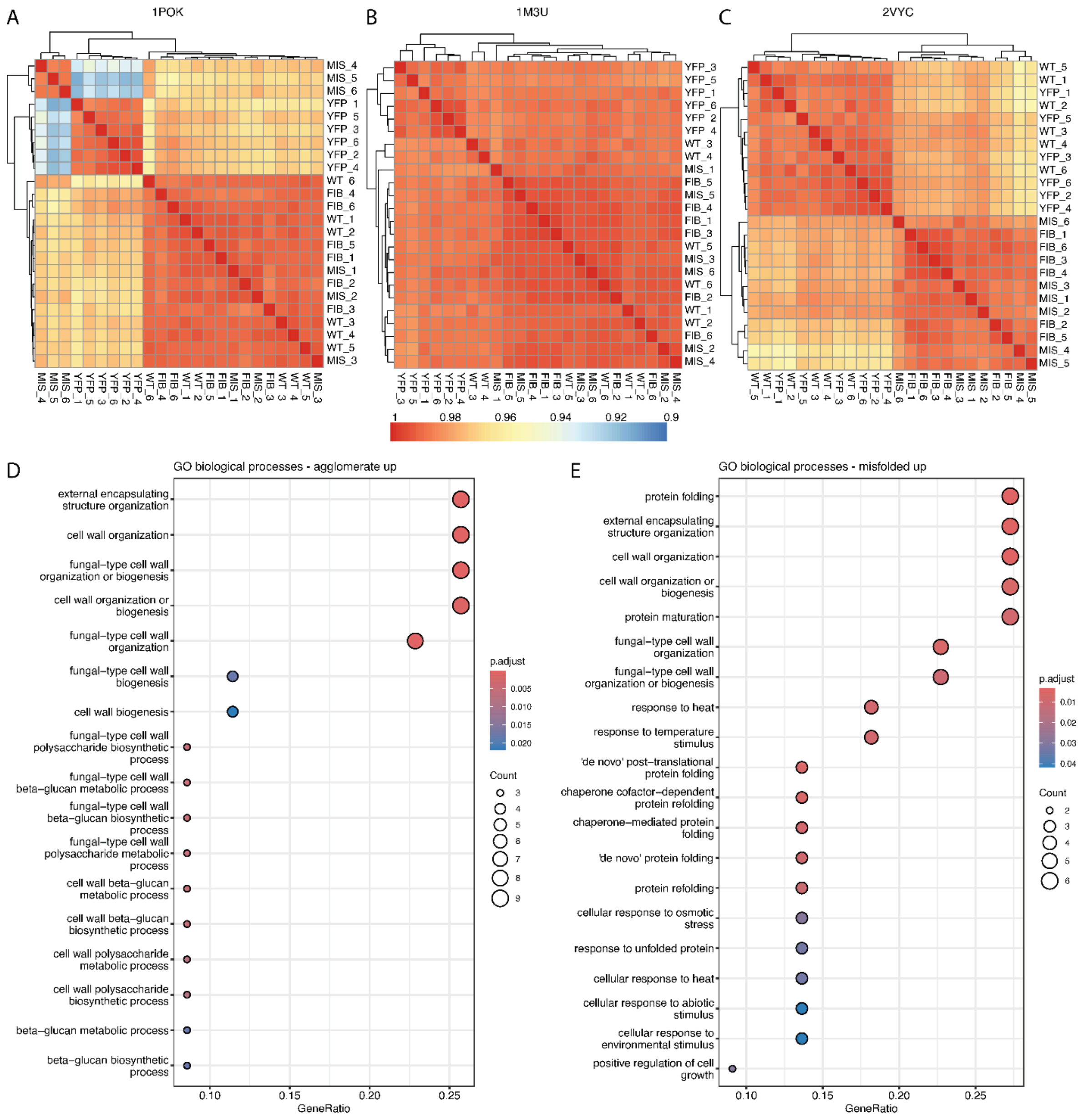

Appendix Figure S10. Proteomics Enrichment.

A-C Correlogram of the proteomics samples. We note that three samples (MIS_4/5/6) showed a distinct correlation pattern, but our conclusions did not change when these were excluded from the analyses. **A.** 1POK samples. **B.** 1M3U samples. **C.** 2VYC samples. **D.** GO enrichment analysis of proteins with a higher abundance in cells expressing the 2VYC agglomerate variant relative to the 2VYC wild type. **E.** Same as D, for 2VYC misfolded compared to 2VYC wild type.

Appendix Table S1. Details of homomers targeted for mutations:

Details of homomers from (Garcia-Seisdedos *et al*, 2017). PDB code, number of subunits, symmetry, ORF, mutated positions. The linker for each protein was GGGGSGGGGS and the fused fluorophore was Venus YFP. The homomer 3N75 was removed from this study due its toxicity in yeast. 1YAC and 1L6W were removed as their filament mutants expressed poorly and cells expressing them showed pronounced autofluorescence.

PDB code	Gene name	Protein name	Ref.	# subunits	MW	Sym	ORF	Agglomerating inducing mutations	Misfolding inducing mutations	Mutations inactivating activity	Used in this study	Reason for discarding
2AN9	gmk	Guanylate kinase	(Hible *et al*, 2005)	6	23.6	D3	2an9-Venus	D60Y/E61Y/K63Y/E64Y	Q108K/I110K/F121K/I122K/L123K & truncation (deletion 3-181bp of 2an9 gene)	No	Yes	
1D7A	purE	N5-carboxyaminoimidazole ribonucleotide mutase	(Mathews *et al*, 1999)	8	16.9	D4	Venus-1d7a	K11L/E22L/E25L/D158L	A123K/G124K/N127K/A128K & G89Stop	No	Yes	
1FRW	mobA	Molybdenum cofactor guanylyltransferase	(Lake *et al*, 2000)	8	21.6	D4	1frw-Venus	D170L/D173L/K175L/D176L	Truncation (deletion 3-271bp of 1frw gene)	No	Yes	
2CG4	asnC	Regulatory protein AsnC	(Thaw *et al*, 2006)	8	17	D4	Venus-2cg4	K126Y/D131Y	C70K/I72K/G73K/I74K & K126Stop	No	Yes	
1YAC	ycaC	Probable hydrolase YcaC	(Colovos *et al*, 1998)	10	22.9	D5	1yac-Venus	D92Y/E94Y/K98Y/K101Y		No	No	Low expression of mutant
1L6W	fsaA	Fructose-6-phosphate aldolase 1	(Thorell *et al*, 2002)	10	23	D5	1l6w-Venus	K97Y/K100Y/E102Y		No	No	Low expression of mutant
2IV1	cynS	Cyanate hydratase	Not published	10	17	D5	2iv1-Venus	K24L/K25L/D26L	Truncation (deletion 3-150bp of 2iv1 gene)	No	Yes	
2VYC	adiA	Biodegradative arginine decarboxylase	(Andréll *et al*, 2009)	10	84.4	D5	2vyc-Venus	K491Y/D494Y/D497Y	Truncation (deletion 3-1005bp of 2vyc gene)	T420A (Hong *et al*, 2021)	Yes	
2WCV	fucU	L-fucose mutarotase	(Lee *et al*, 2009)	10	15.5	D5	2wcv-Venus	E77Y	Truncation (deletion 3-153bp of 2wcv gene)	No	No	Low frequency of filaments
1POK	iadA	Isoaspartyl dipeptidase	(Jozic *et al*, 2003)	8	41.1	D4	Venus-1pok	E239Y & E239Y/E243Y/K247Y	L354K/L356K/V357K/M358K & L240Stop	R169M (Kime *et al*, 2015)	Yes	
1M3U	panB	3-methyl-2-oxobutanoate hydroxymethyltransferase	(von Delft *et al*, 2003)	10	28.2	D5	1m3u-Venus	D157L/E158L/D161L	L180K/V183K/A188K/I191K<br>Truncation (deletion 3-468bp of 1m3u gene)	D45A/D84A/E114A (von Delft *et al*, 2003)	Yes	
3N75	ldcI	Lysine Decarboxylase	(Kanjee *et al*, 2011)	10	81.2	D5	3n75-Venus	D460L		No	No	Toxic for the cell

Appendix Table S2. Manual quantifications of agglomerates in dividing cells.

Protein	n	%
Filaments passing from mother to daughter		
1pok	83	30.1
1m3u	69	20.2
2vyc	59	20.3
Filament aligned with tubulin		
1pok	84	59.5
1m3u	56	46.4
2vyc	48	54.1

Appendix Table S3. Summary of evidence towards an agglomerate or an aggregated state for the different constructs.

We observed a consistent behavior of the different constructs across various experimental methodologies. For circular dichroism, we signal with a "+ or "-" constructs that maintain or lose their wild type secondary structure content. Some constructs show an intermediate behavior and are reported as "o". For the hybrid-binding experiment or "dissolution with WT experiment" (related to Fig. 2), "+" or "-" denote constructs that are dissolved or not dissolved by expressing the WT. Two constructs show an intermediate behavior and are reported as "o". For the expression level, we signal with a "+ or "-" constructs that exhibit high or low expression. Two constructs show an intermediate expression and are reported as "o". For the "co-localization with chaperones" experiment, "+" and "-" denote constructs that do, or do not colocalize with chaperones. Three constructs show intermediate co-localization patterns and are reported as "o". Finally, for the pull-down experiment we report as "+" and "-" constructs that do, or do not, pull down associated chaperones.

	**Behave consistently as agglomerates**				**Behave consistently as aggregates**								**Behave partially as agglomerates and partially as aggregates**			
	1pok	1m3u	2cg4	2vyc	1frw	2vi1	1pok-mis	1m3u-mis	2cg4-mis	2vyc-trunc	1frw-mis	2vi1-trunc	2an9	1d7a	2an9-mis	1d7a-mis
**Circular dichroism**	+	+	+	+	-	N/A (insoluble)	not tested	not tested	not tested	not tested	not tested	not tested	o	-	not tested	not tested
**Hybrid binding (dissolution with WT)**	+	+	+	+	-	-	-	-	-	-	-	-	o	o	-	-
**Expression level (microscopy)**	+	+	+	+	-	-	-	-	-	-	-	-	+	+	o	o
**co-localization with chaperones**	-	-	-	o	+	+	o	+	N/A (low expression)	+	N/A (low expression)	N/A (low expression)	o	+	+	+
**chaperones pulled-down with construct**	-	-	not tested	not tested	not tested	not tested	+	+	not tested	not tested	not tested	not tested	not tested	not tested	not tested	not tested

Appendix References

Andréll J, Hicks MG, Palmer T, Carpenter EP, Iwata S & Maher MJ (2009) Crystal structure of the acid-induced arginine decarboxylase from Escherichia coli: reversible decamer assembly controls enzyme activity. *Biochemistry* 48: 3915–3927

Colovos C, Cascio D & Yeates TO (1998) The 1.8 A crystal structure of the ycaC gene product from Escherichia coli reveals an octameric hydrolase of unknown specificity. *Structure* 6: 1329–1337

von Delft F, Inoue T, Saldanha SA, Ottenhof HH, Schmitzberger F, Birch LM, Dhanaraj V, Witty M, Smith AG, Blundell TL, *et al* (2003) Structure of E. coli ketopantoate hydroxymethyl transferase complexed with ketopantoate and Mg2+, solved by locating 160 selenomethionine sites. *Structure* 11: 985–996

Garcia-Seisdedos H, Empereur-Mot C, Elad N & Levy ED (2017) Proteins evolve on the edge of supramolecular self-assembly. *Nature* 548: 244–247

Hible G, Renault L, Schaeffer F, Christova P, Zoe Radulescu A, Evrin C, Gilles A-M & Cherfils J (2005) Calorimetric and crystallographic analysis of the oligomeric structure of Escherichia coli GMP kinase. *J Mol Biol* 352: 1044–1059

Hong EY, Lee S-G, Yun H & Kim B-G (2021) Improving the stability and activity of arginine decarboxylase at alkaline pH for the production of agmatine. *Front Catal* 1

Jozic D, Kaiser JT, Huber R, Bode W & Maskos K (2003) X-ray structure of isoaspartyl dipeptidase from E.coli: a dinuclear zinc peptidase evolved from amidohydrolases. *J Mol Biol* 332: 243–256

Kanjee U, Gutsche I, Alexopoulos E, Zhao B, El Bakkouri M, Thibault G, Liu K, Ramachandran S, Snider J, Pai EF, *et al* (2011) Linkage between the bacterial acid stress and stringent responses: the structure of the inducible lysine decarboxylase. *EMBO J* 30: 931–944

Kime L, Vincent HA, Gendoo DMA, Jourdan SS, Fishwick CWG, Callaghan AJ & McDowall KJ (2015) The first small-molecule inhibitors of members of the ribonuclease E family. *Sci Rep* 5: 8028

Lake MW, Temple CA, Rajagopalan KV & Schindelin H (2000) The crystal structure of the Escherichia coli MobA protein provides insight into molybdopterin guanine dinucleotide biosynthesis. *J Biol Chem* 275: 40211–40217

Lee K-H, Ryu K-S, Kim M-S, Suh H-Y, Ku B, Song Y-L, Ko S, Lee W & Oh B-H (2009) Crystal structures and enzyme mechanisms of a dual fucose mutarotase/ribose pyranase. *J Mol Biol* 391: 178–191

Mathews II, Kappock TJ, Stubbe J & Ealick SE (1999) Crystal structure of Escherichia coli PurE, an unusual mutase in the purine biosynthetic pathway. *Structure* 7: 1395–1406

Thaw P, Sedelnikova SE, Muranova T, Wiese S, Ayora S, Alonso JC, Brinkman AB, Akerboom J, van der Oost J & Rafferty JB (2006) Structural insight into gene transcriptional regulation and effector binding by the Lrp/AsnC family. *Nucleic Acids Res* 34: 1439–1449

Thorell S, Schürmann M, Sprenger GA & Schneider G (2002) Crystal structure of decameric fructose-6-phosphate aldolase from Escherichia coli reveals inter-subunit helix swapping as a structural basis for assembly differences in the transaldolase family. *J Mol Biol* 319: 161–171
