# Supplementary figures and images for: Mutation-induced filaments of folded proteins are inert and non-toxic in a cellular system
Source: Mol Syst Biol. 2025 Sep 15;21(10):1306–24. doi: 10.1038/s44320-025-00144-y (PMC12494878; doi:10.1038/s44320-025-00144-y)

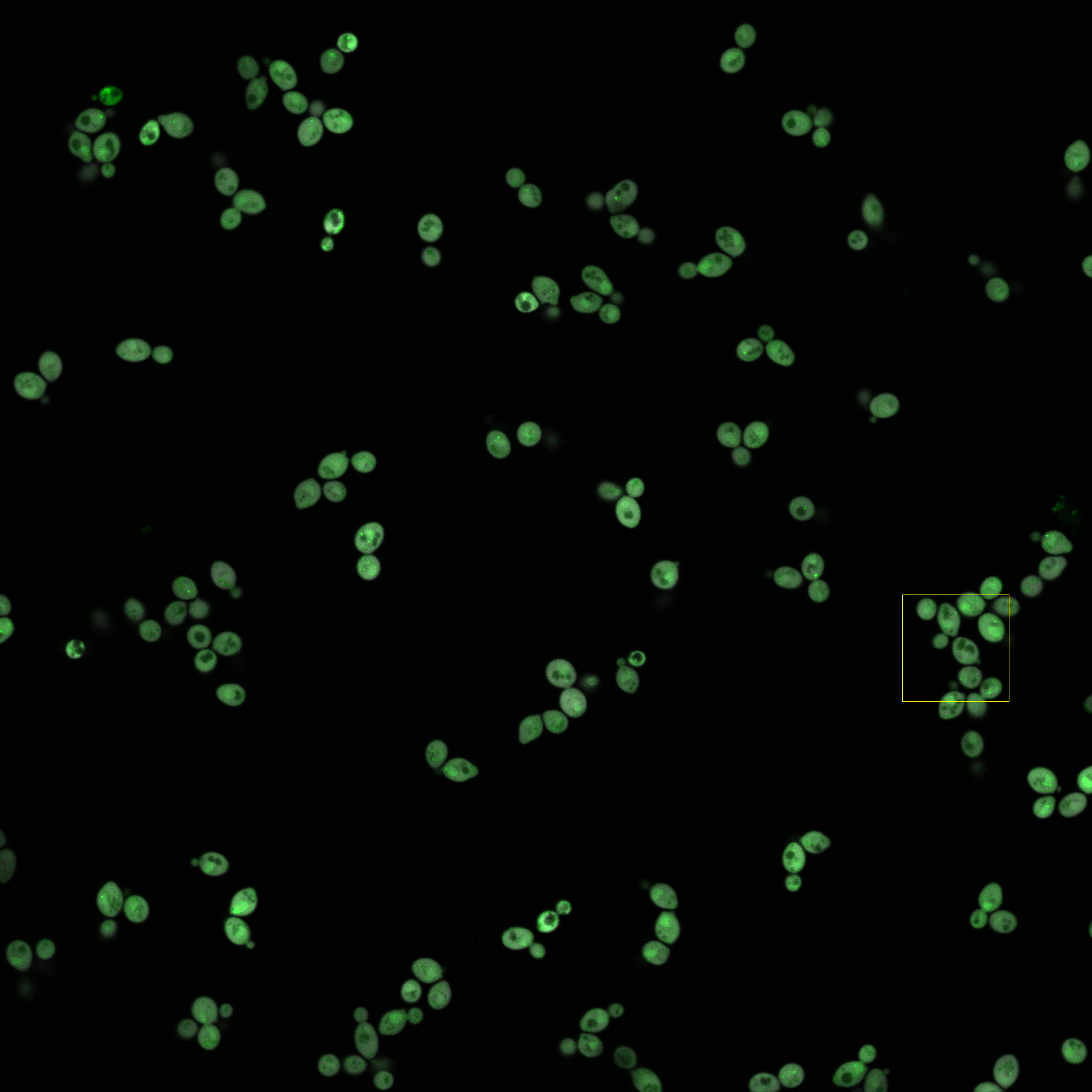

Supplement: Supplementary file 8 — Source data Fig. 1 [file 44320_2025_144_MOESM8_ESM.zip › Fig1/1A/1frw.tif]

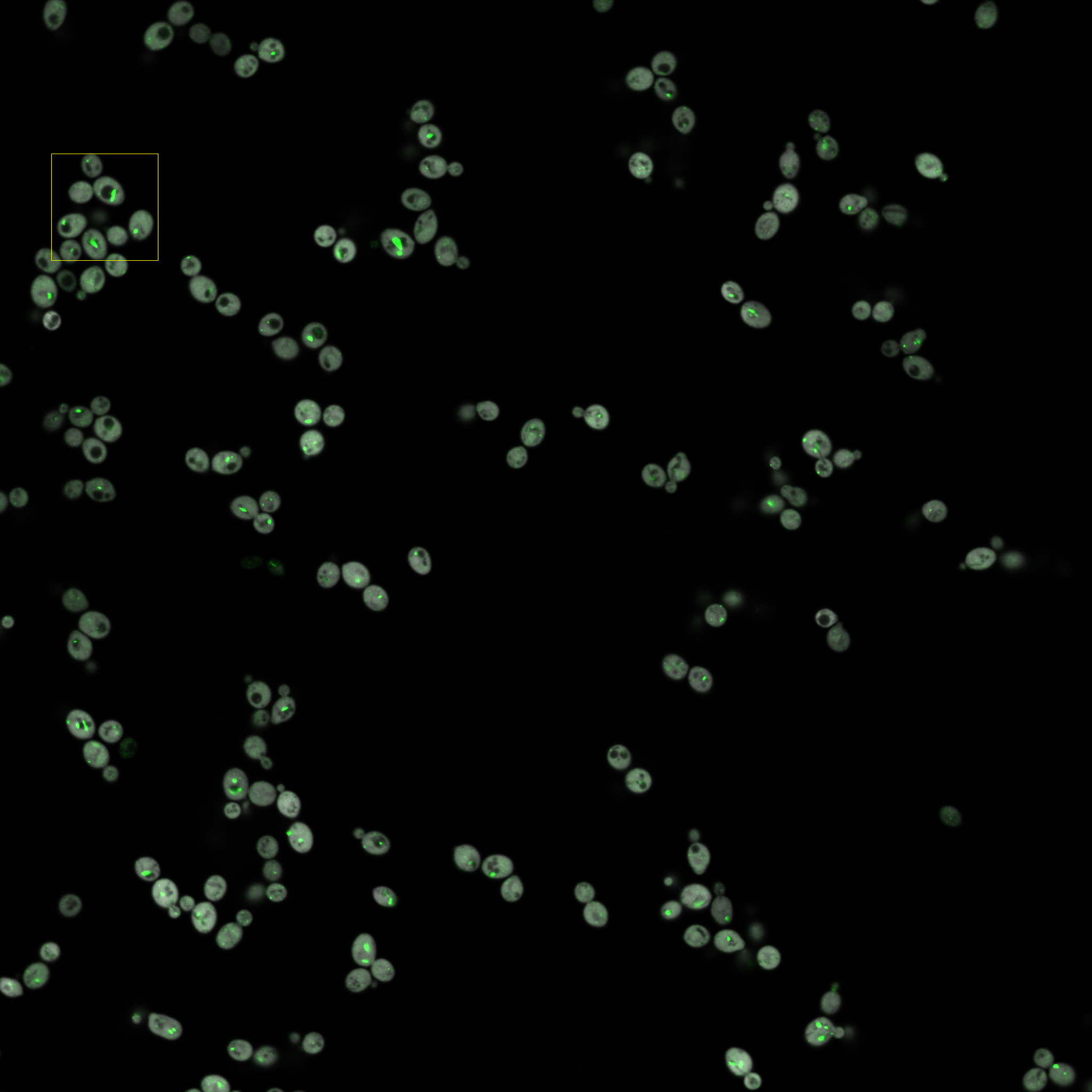

Supplement: Supplementary file 8 — Source data Fig. 1 [file 44320_2025_144_MOESM8_ESM.zip › Fig1/1A/1m3u.tif]

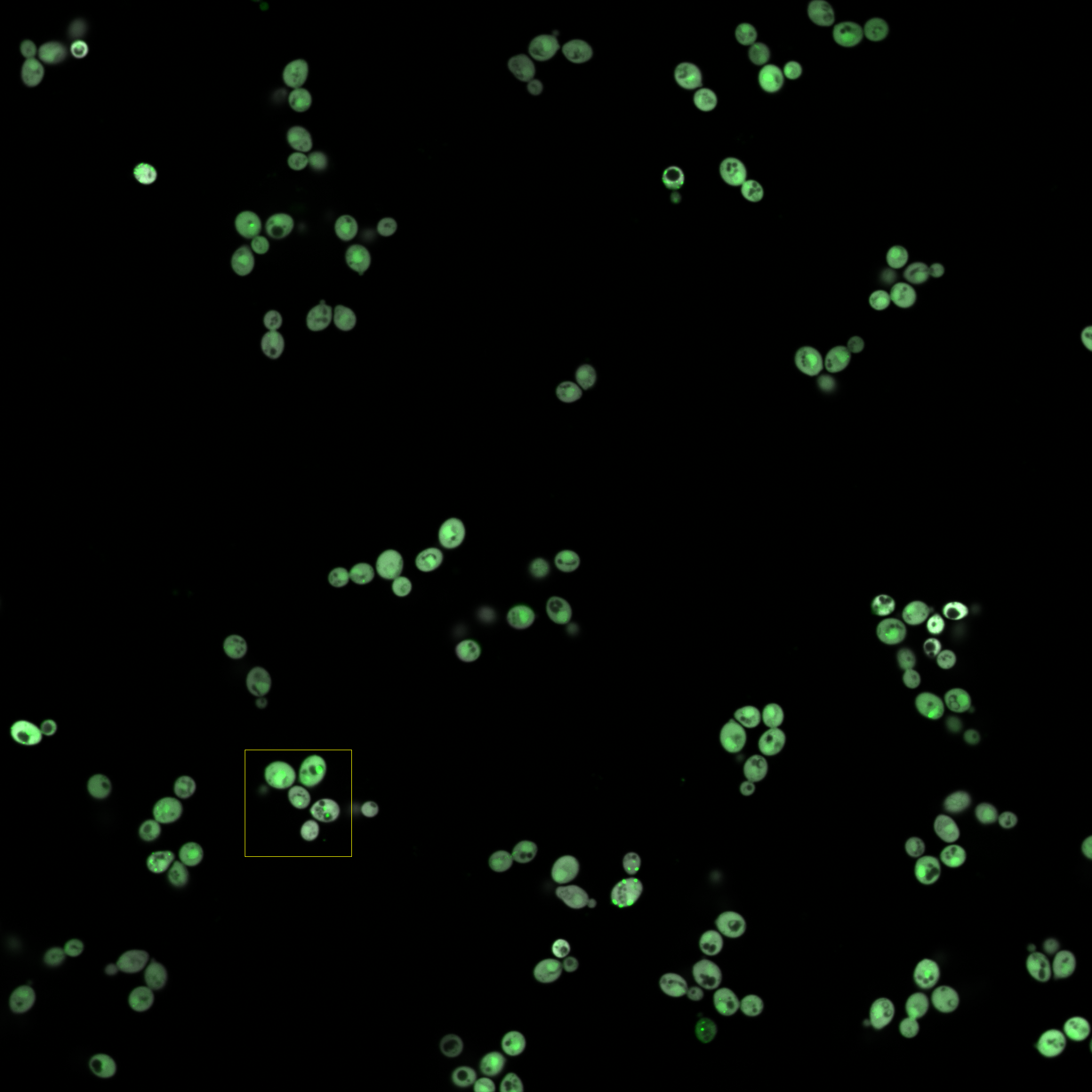

Supplement: Supplementary file 8 — Source data Fig. 1 [file 44320_2025_144_MOESM8_ESM.zip › Fig1/1A/1d7a.tif]

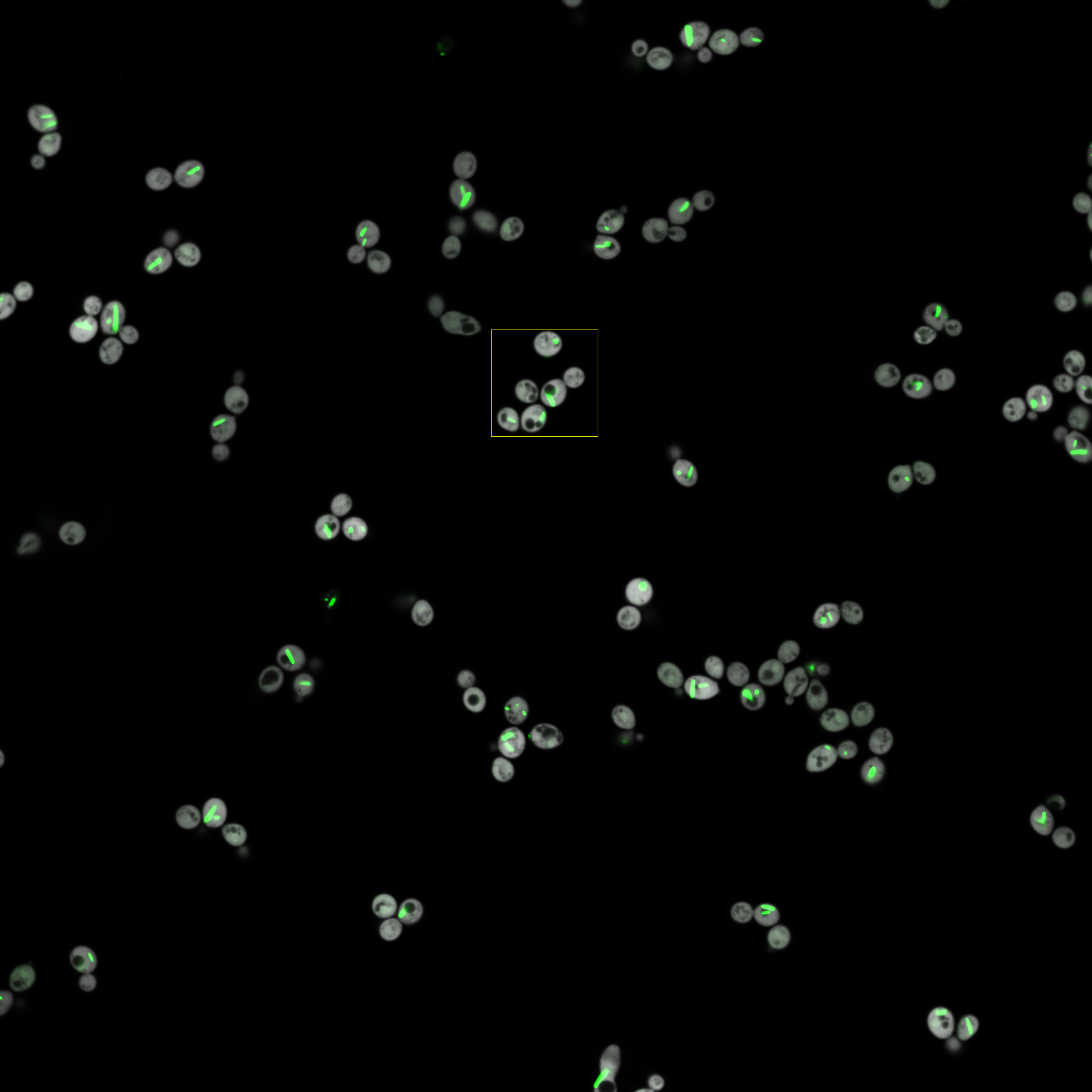

Supplement: Supplementary file 8 — Source data Fig. 1 [file 44320_2025_144_MOESM8_ESM.zip › Fig1/1A/2vyc.tif]

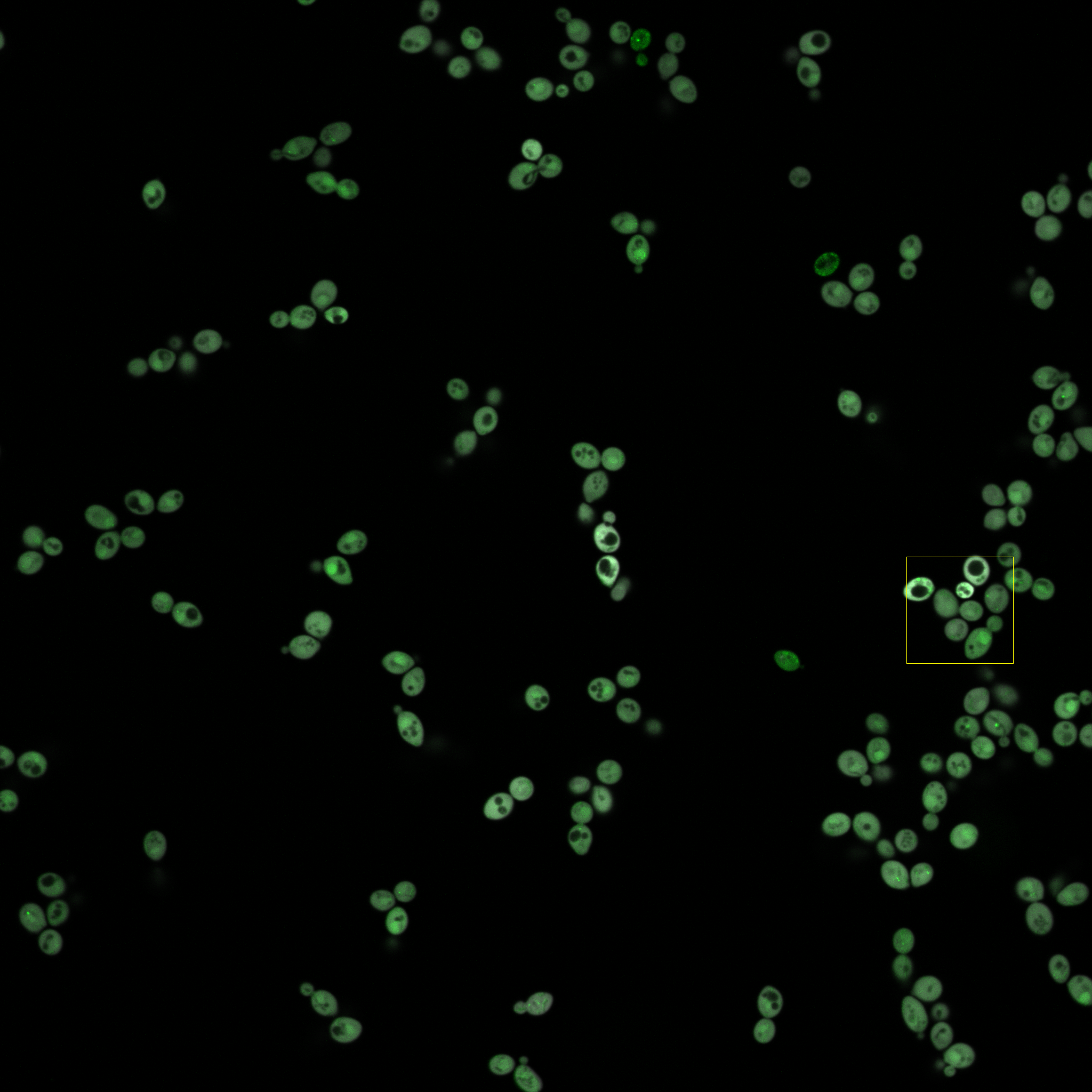

Supplement: Supplementary file 8 — Source data Fig. 1 [file 44320_2025_144_MOESM8_ESM.zip › Fig1/1A/2iv1.tif]

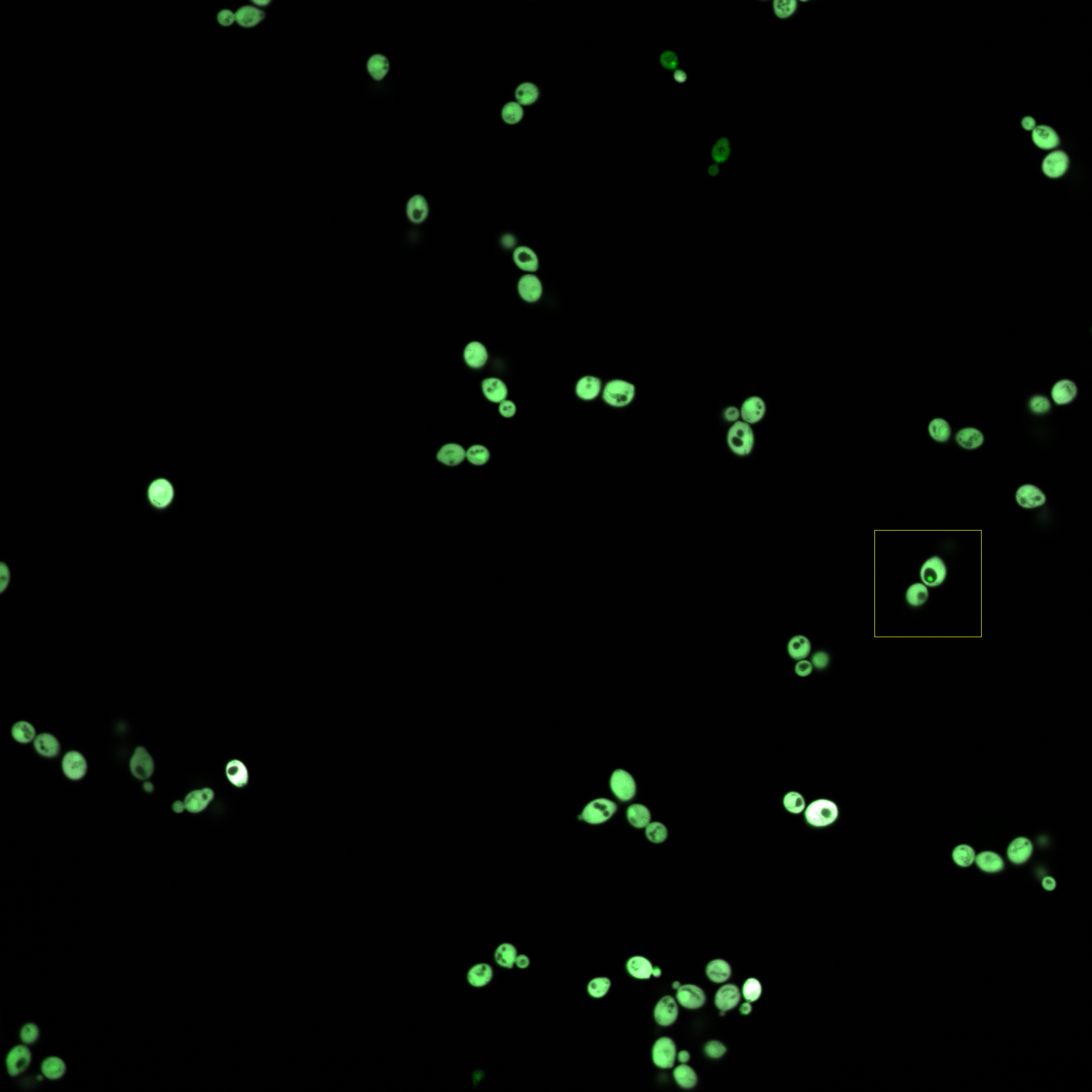

Supplement: Supplementary file 8 — Source data Fig. 1 [file 44320_2025_144_MOESM8_ESM.zip › Fig1/1A/2an9.tif]

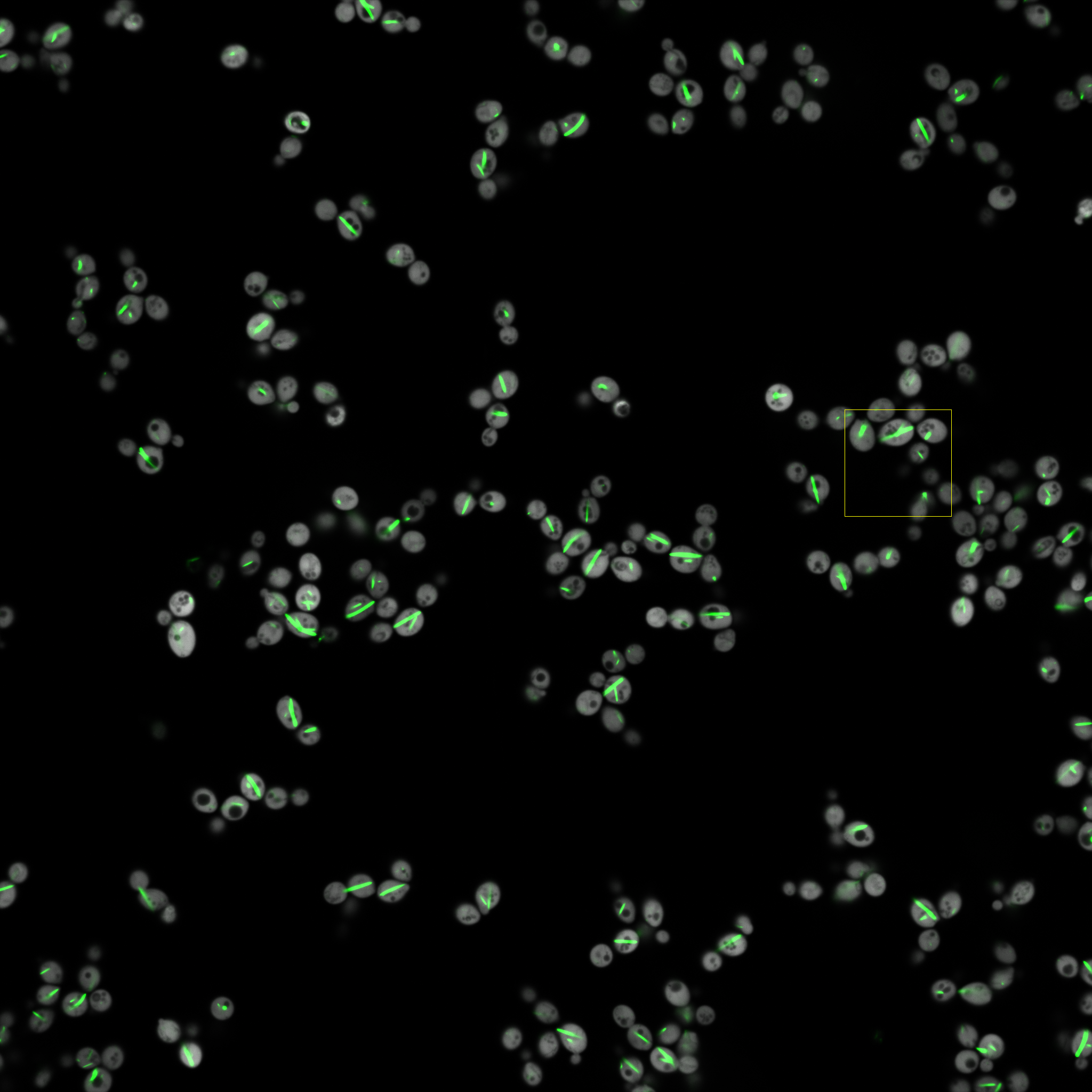

Supplement: Supplementary file 8 — Source data Fig. 1 [file 44320_2025_144_MOESM8_ESM.zip › Fig1/1A/1pok.tif]

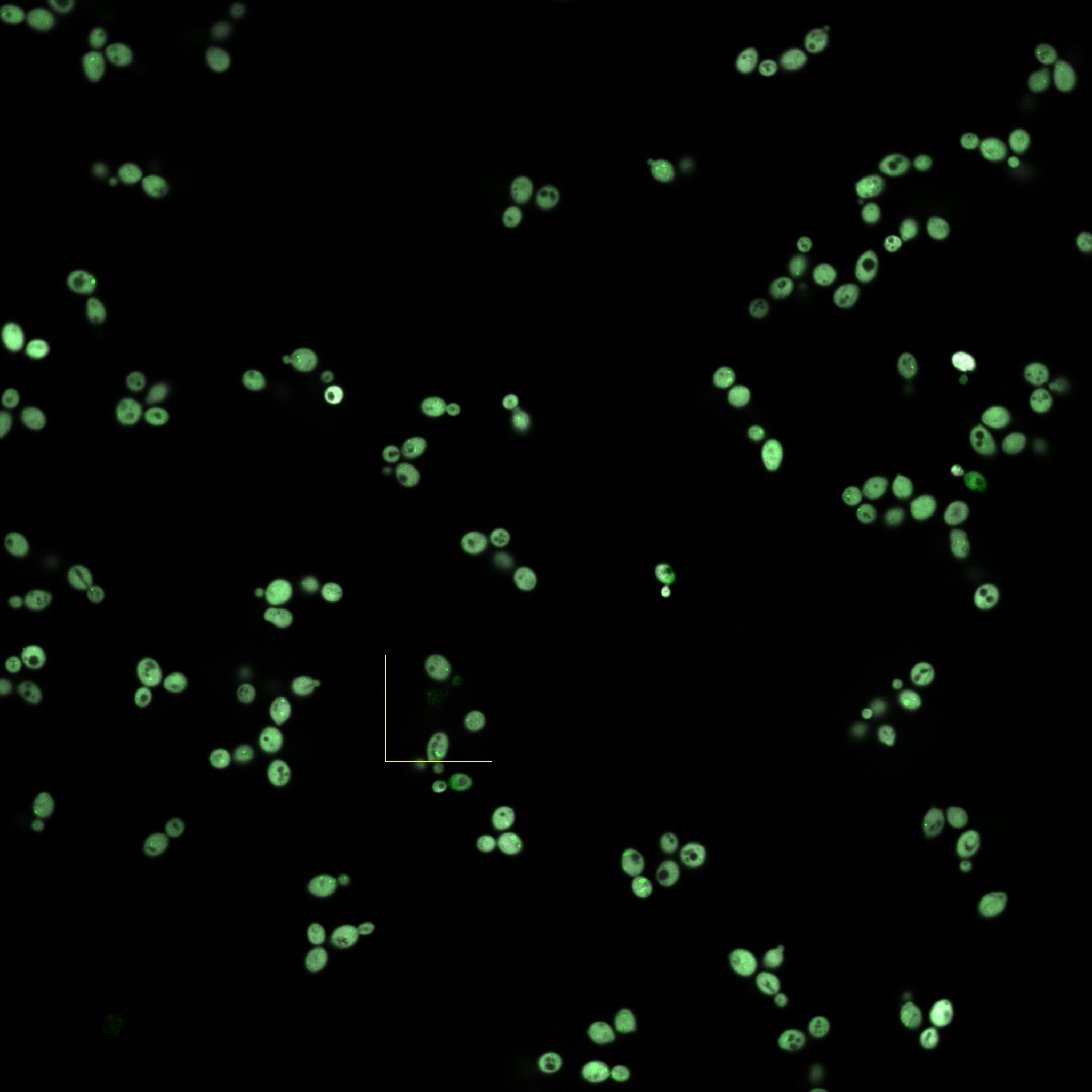

Supplement: Supplementary file 8 — Source data Fig. 1 [file 44320_2025_144_MOESM8_ESM.zip › Fig1/1A/2cg4.tif]

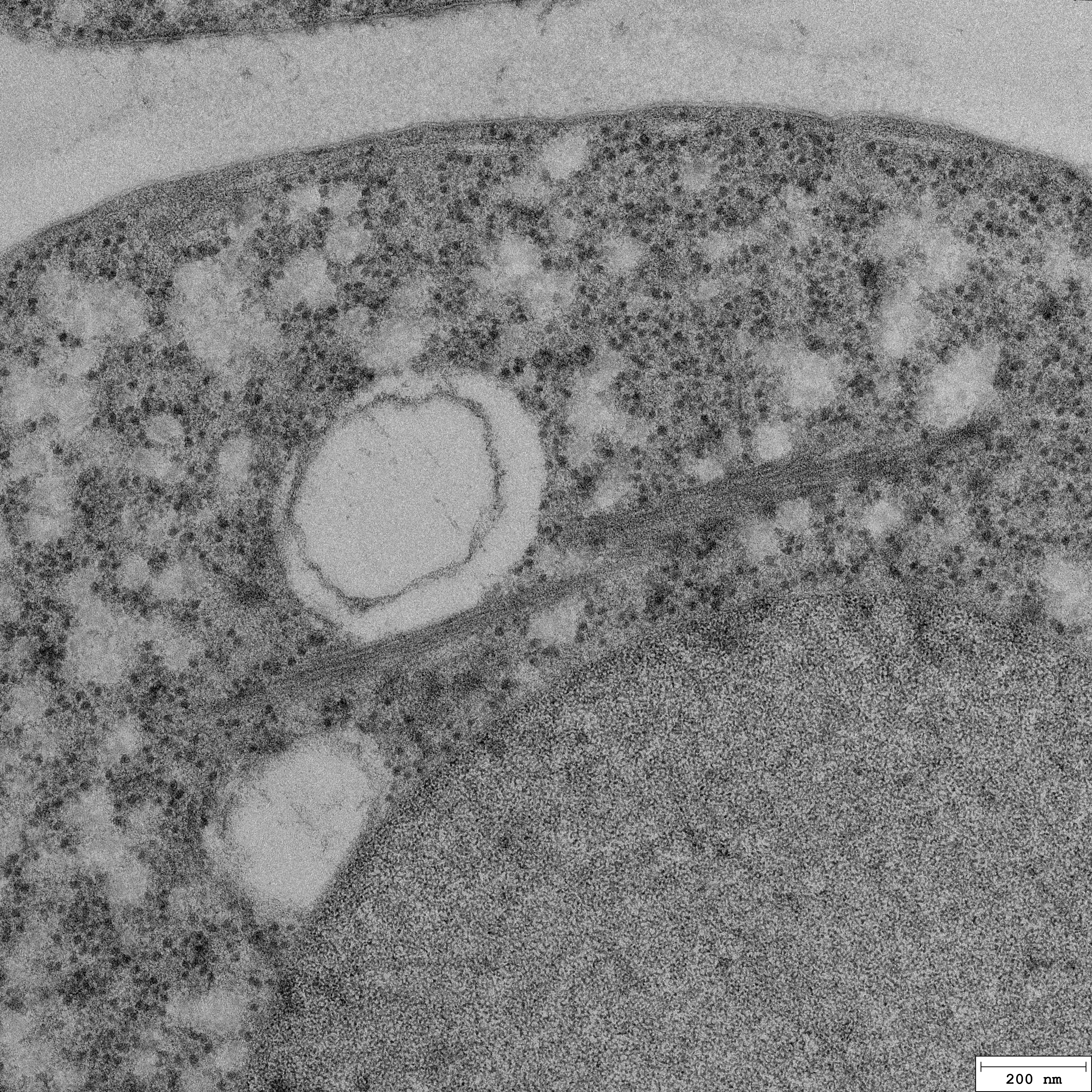

Supplement: Supplementary file 8 — Source data Fig. 1 [file 44320_2025_144_MOESM8_ESM.zip › Fig1/1B/1B_left.tif]

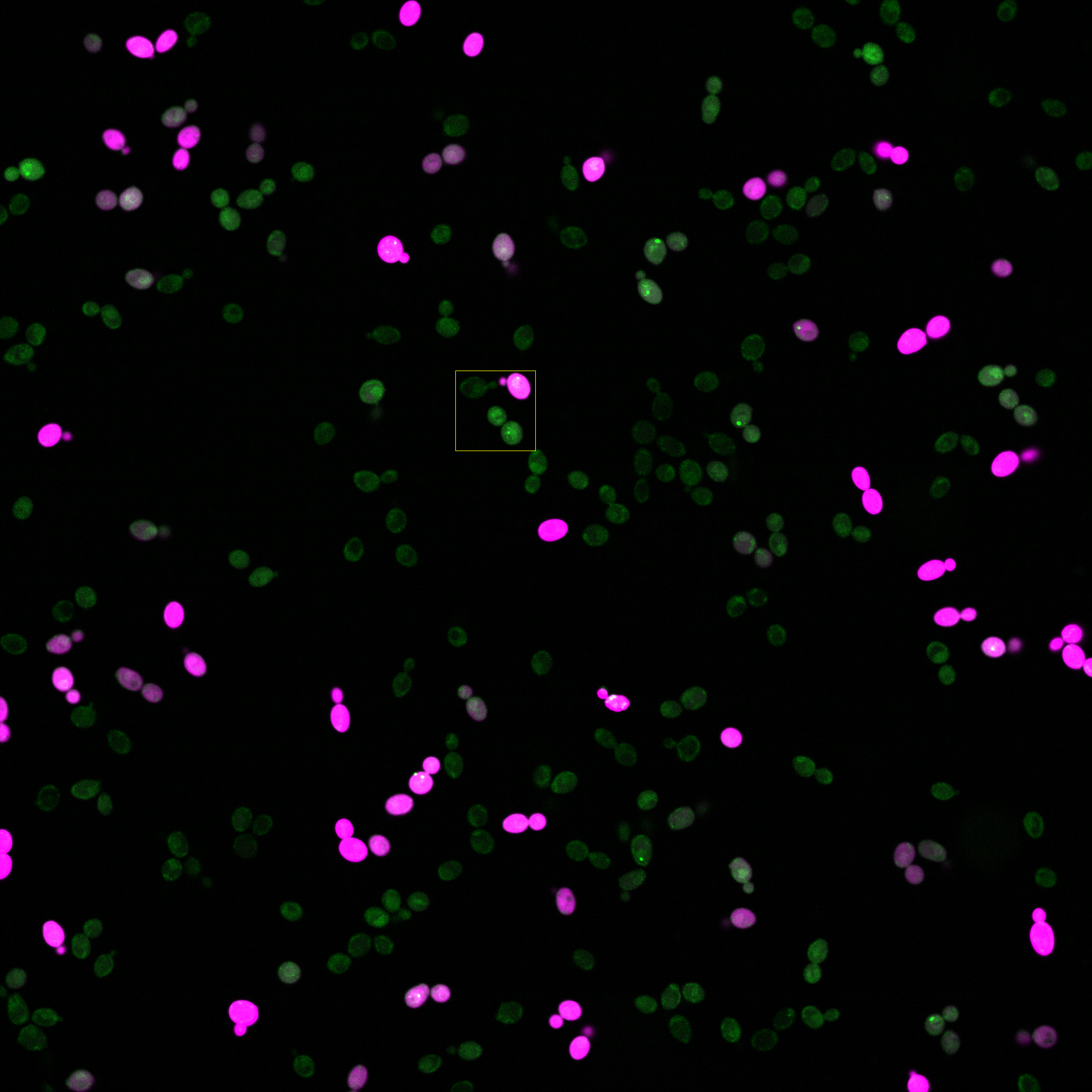

Supplement: Supplementary file 9 — Source data Fig. 2 [file 44320_2025_144_MOESM9_ESM.zip › Fig2/2C/1frw_gal.tif]

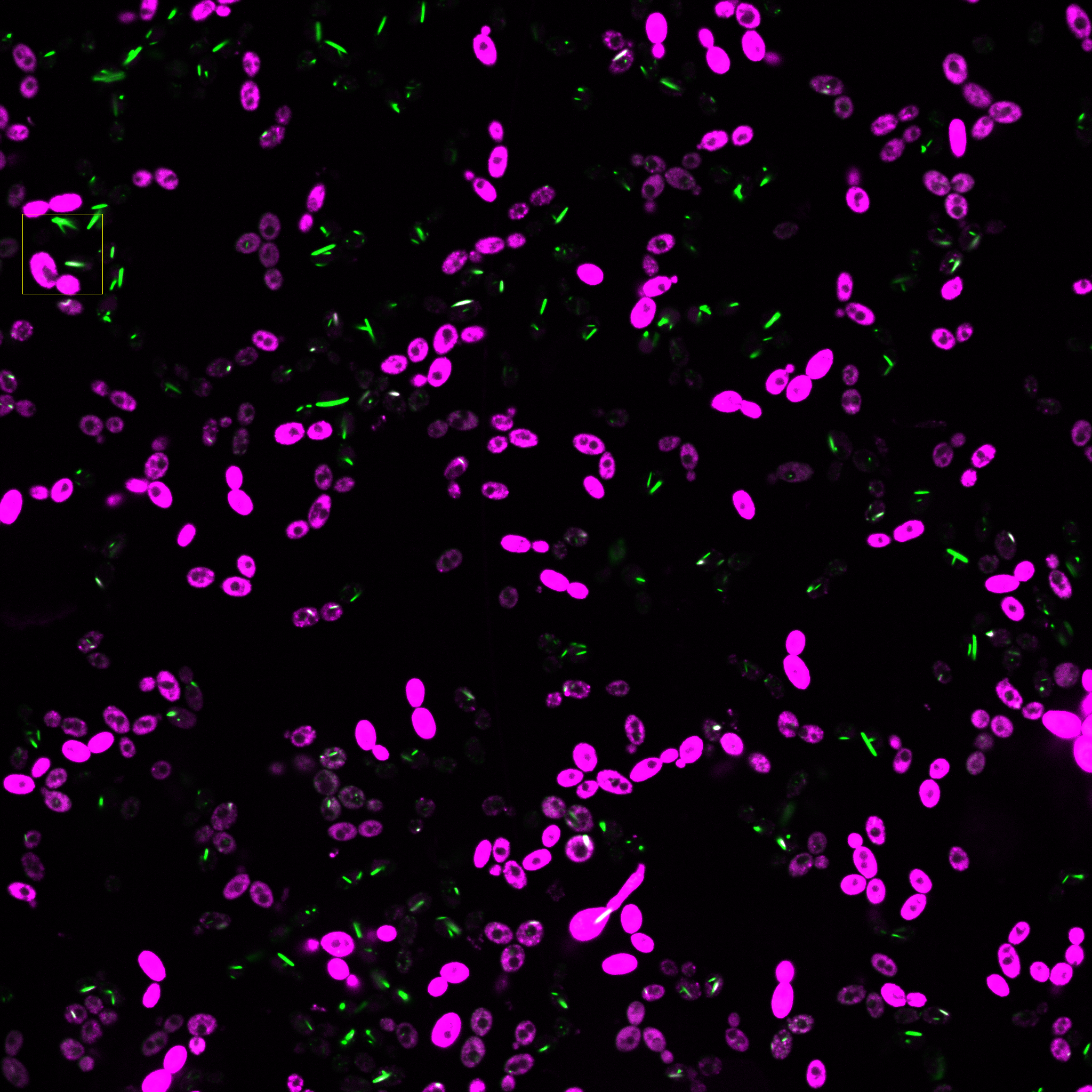

Supplement: Supplementary file 9 — Source data Fig. 2 [file 44320_2025_144_MOESM9_ESM.zip › Fig2/2B/1pok_gal.tif]

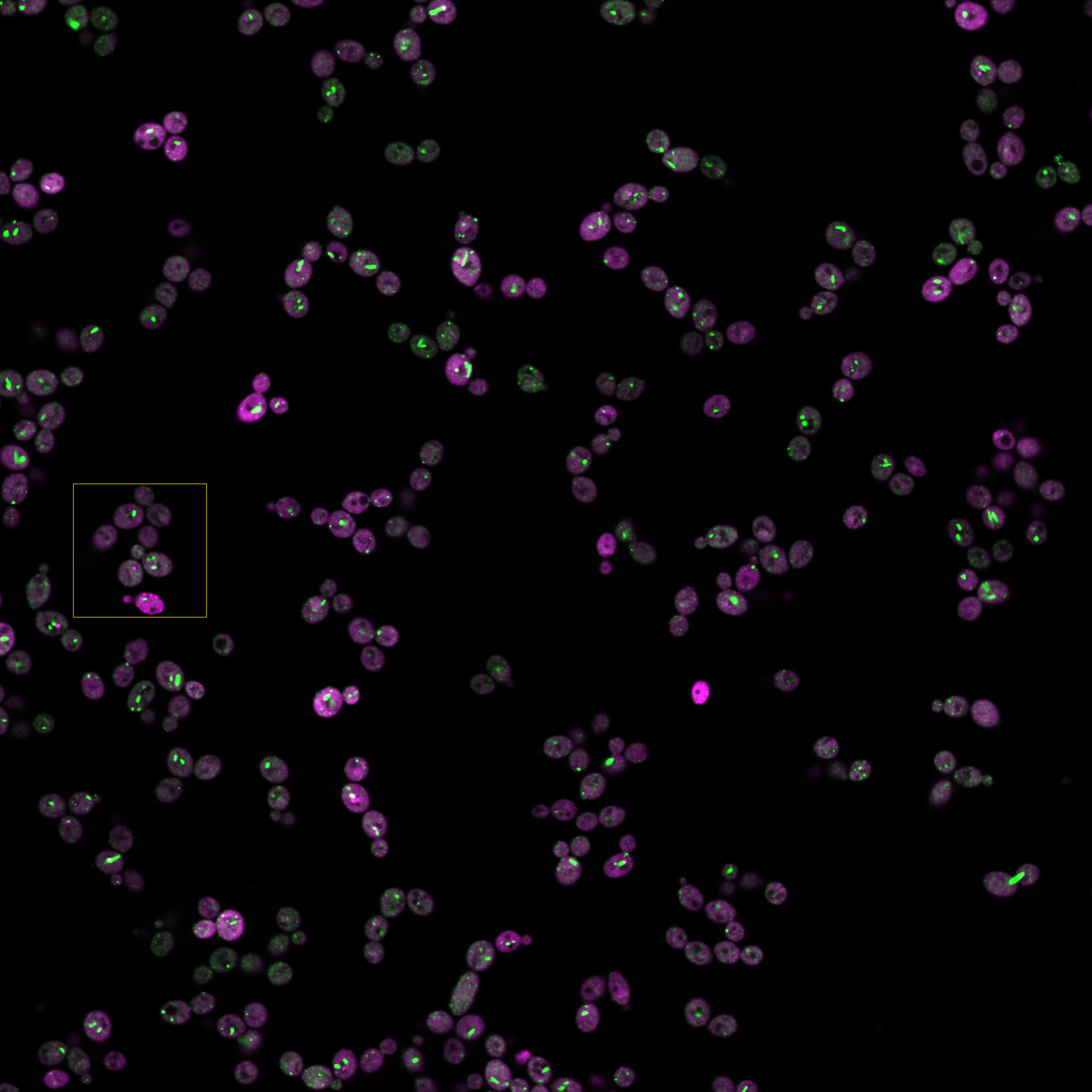

Supplement: Supplementary file 10 — Source data Fig. 3 [file 44320_2025_144_MOESM10_ESM.zip › Fig3/3B/1m3u-hsp104.tif]

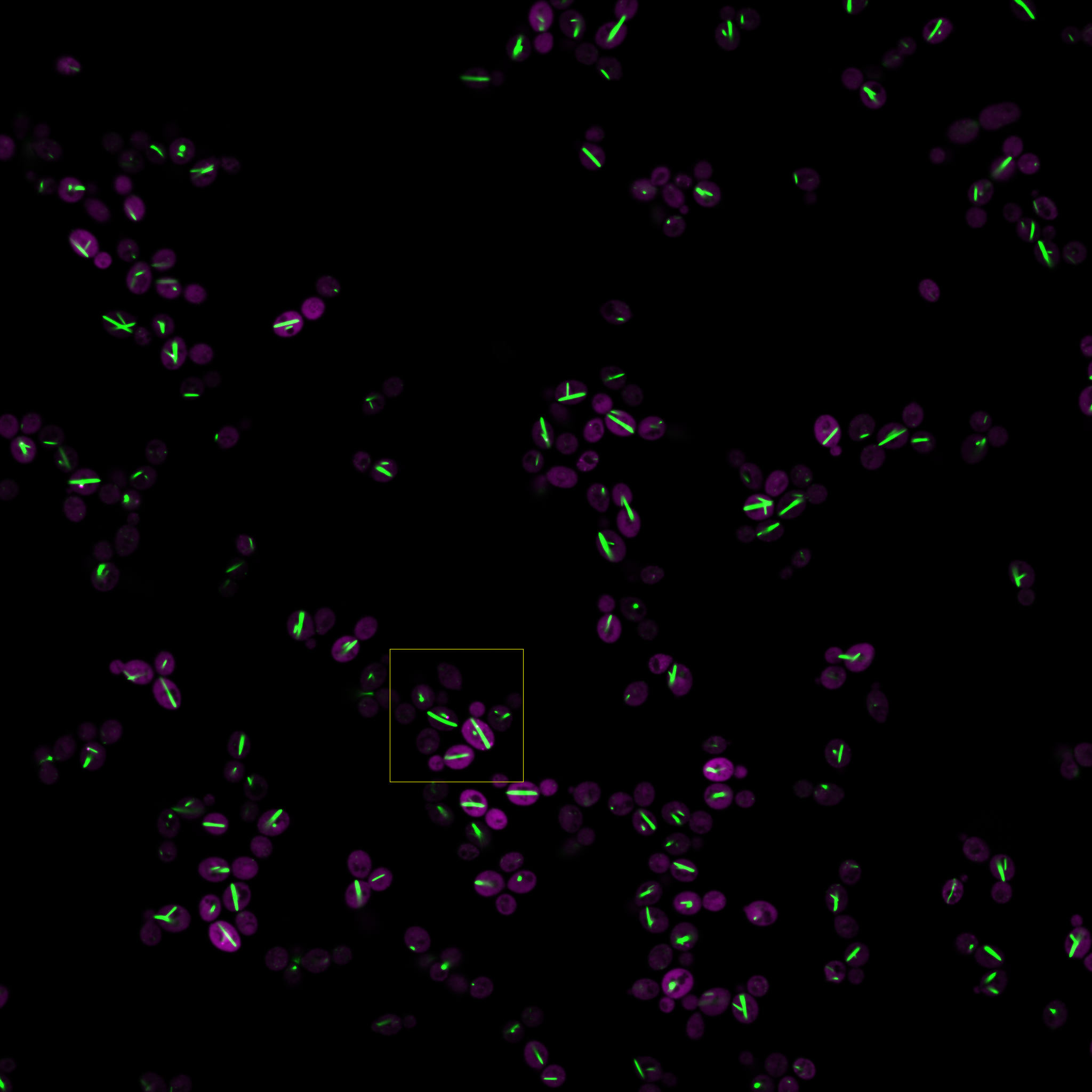

Supplement: Supplementary file 10 — Source data Fig. 3 [file 44320_2025_144_MOESM10_ESM.zip › Fig3/3B/1pok-hsp104.tif]

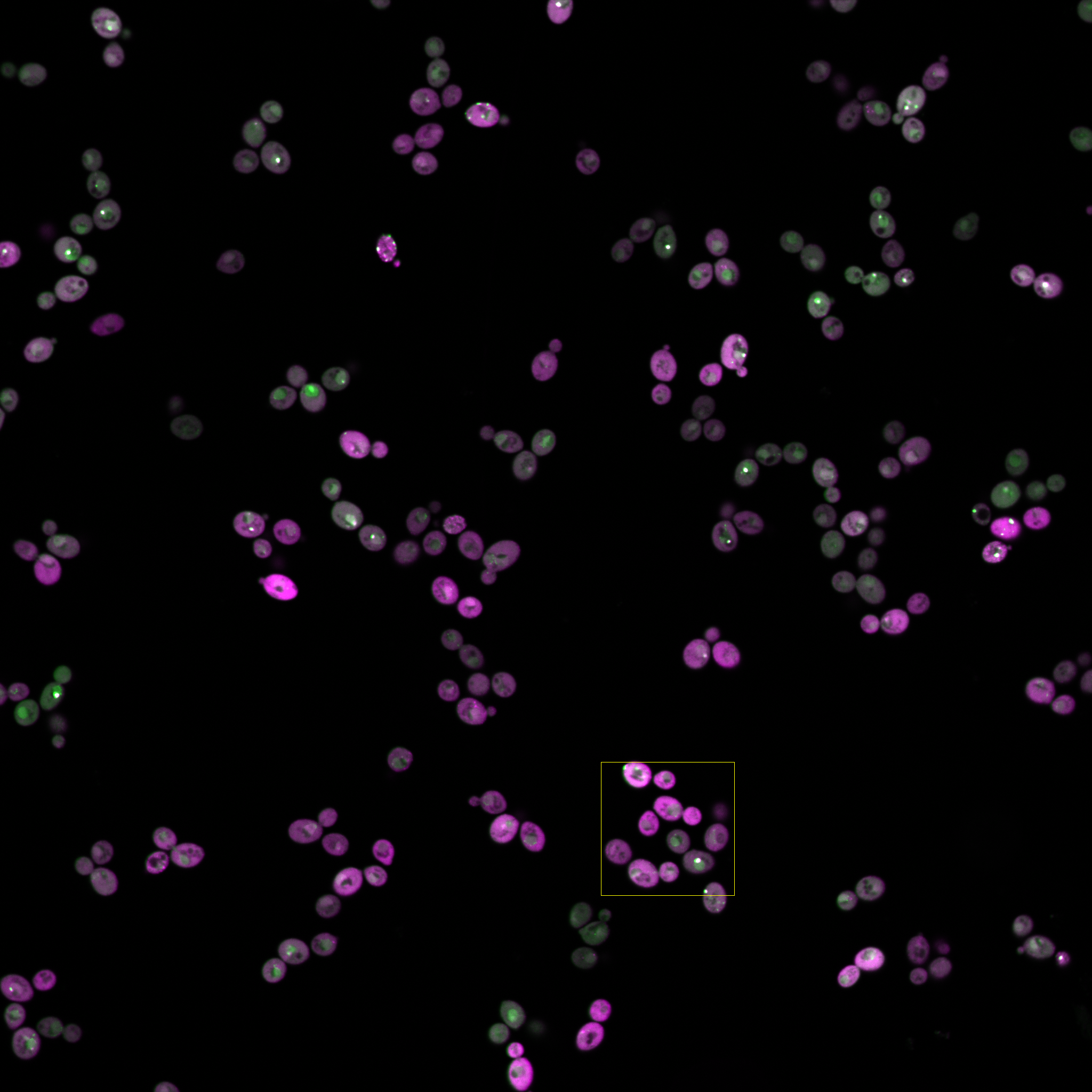

Supplement: Supplementary file 10 — Source data Fig. 3 [file 44320_2025_144_MOESM10_ESM.zip › Fig3/3B/1d7a-hsp104.tif]

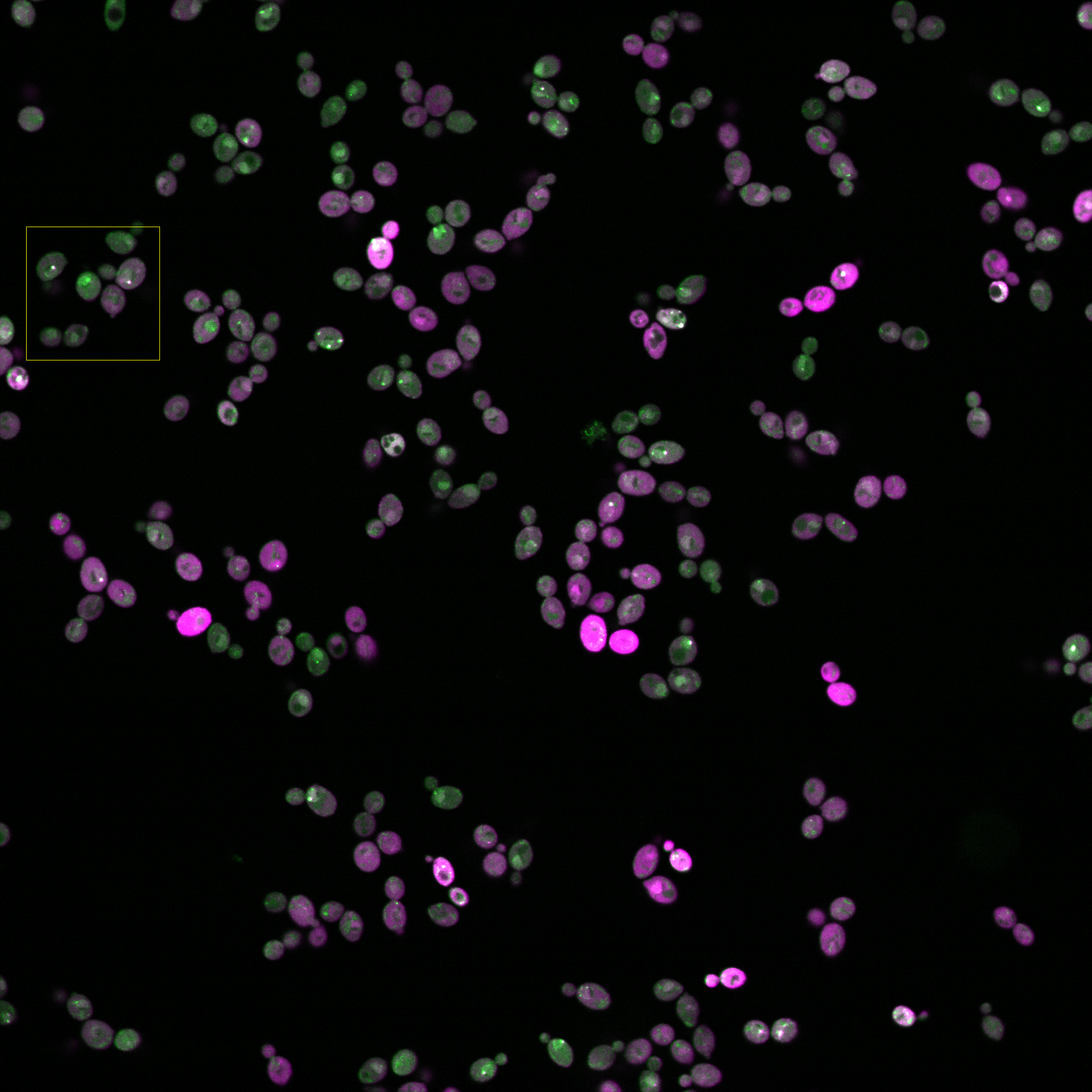

Supplement: Supplementary file 10 — Source data Fig. 3 [file 44320_2025_144_MOESM10_ESM.zip › Fig3/3B/1frw-hsp104.tif]

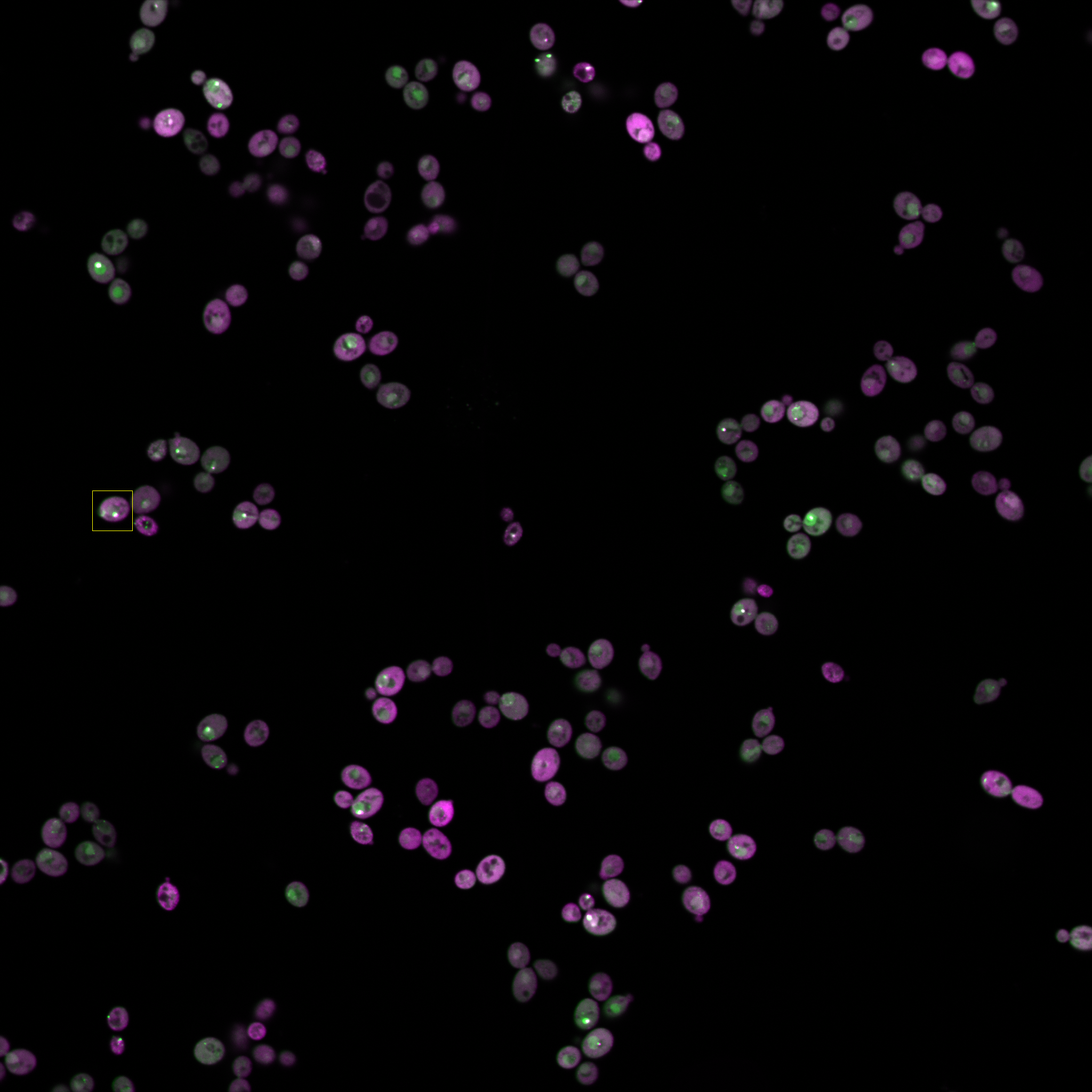

Supplement: Supplementary file 10 — Source data Fig. 3 [file 44320_2025_144_MOESM10_ESM.zip › Fig3/3A/0-49-example.tif]

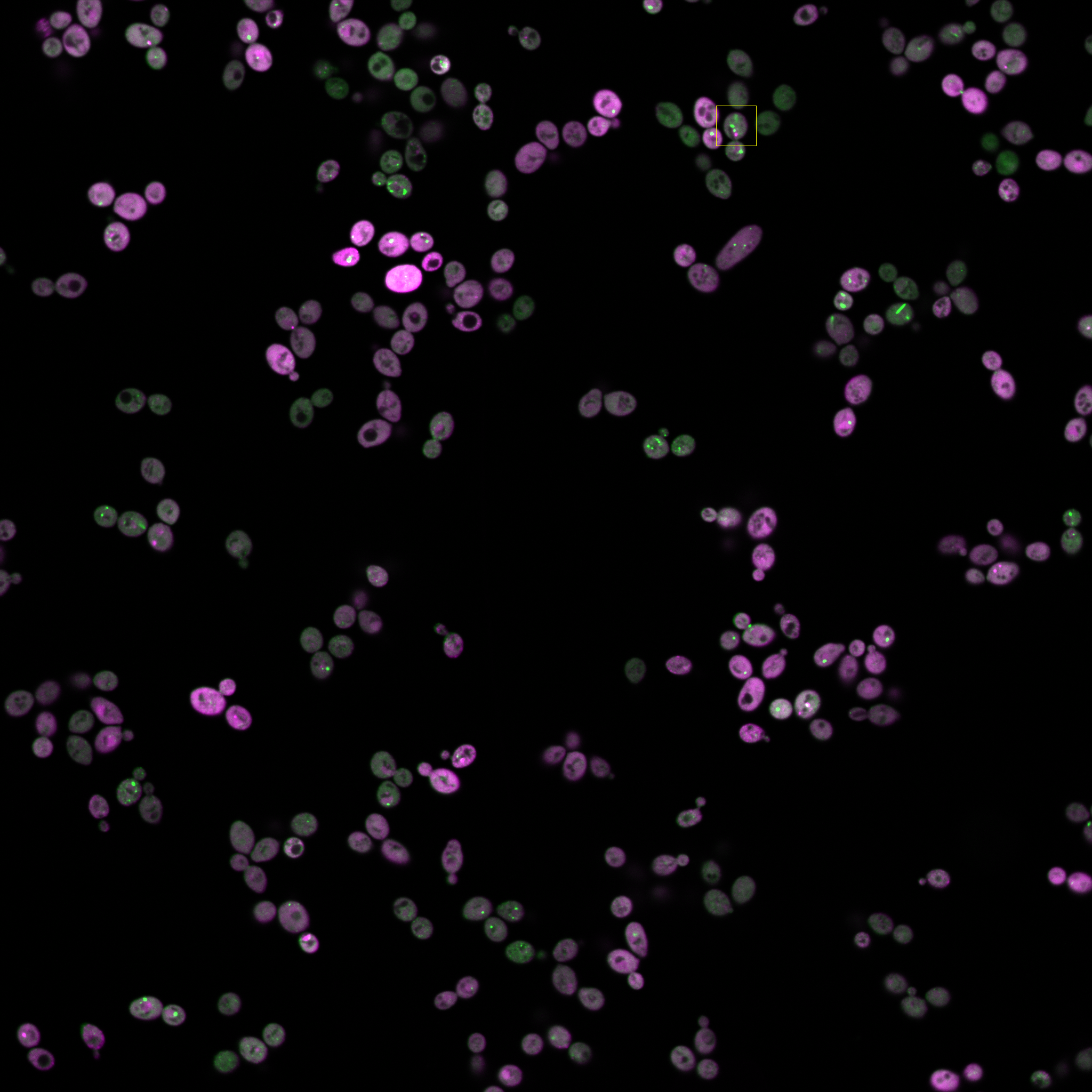

Supplement: Supplementary file 10 — Source data Fig. 3 [file 44320_2025_144_MOESM10_ESM.zip › Fig3/3A/0-24-example.tif]

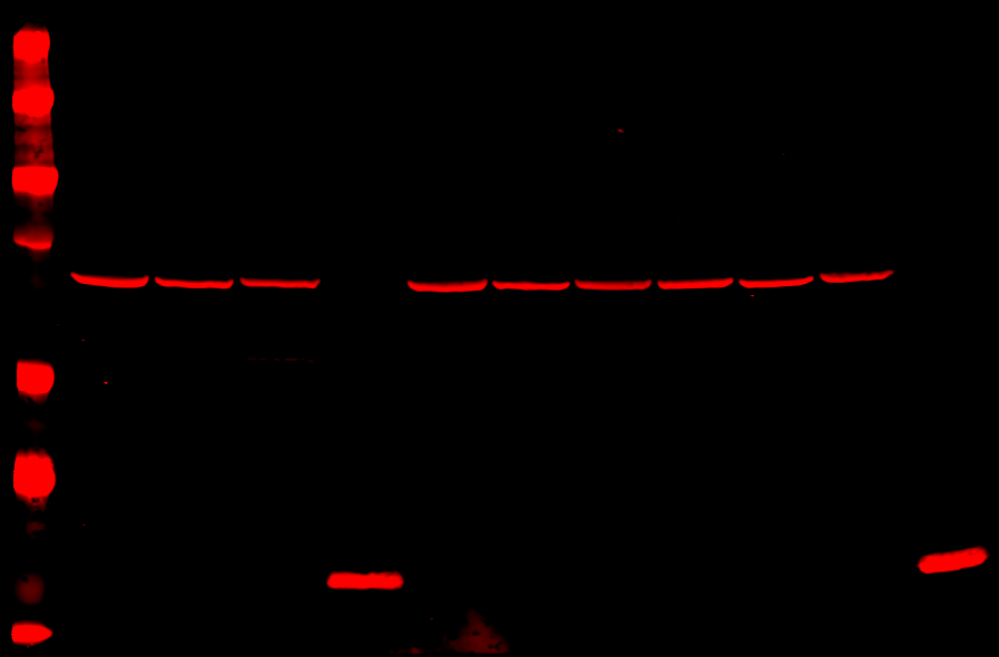

Supplement: Supplementary file 11 — Source data Fig. 4 [file 44320_2025_144_MOESM11_ESM.zip › Fig4/4B/20231128_1POK_new_no_MG_Ub_yfp.png.tif]

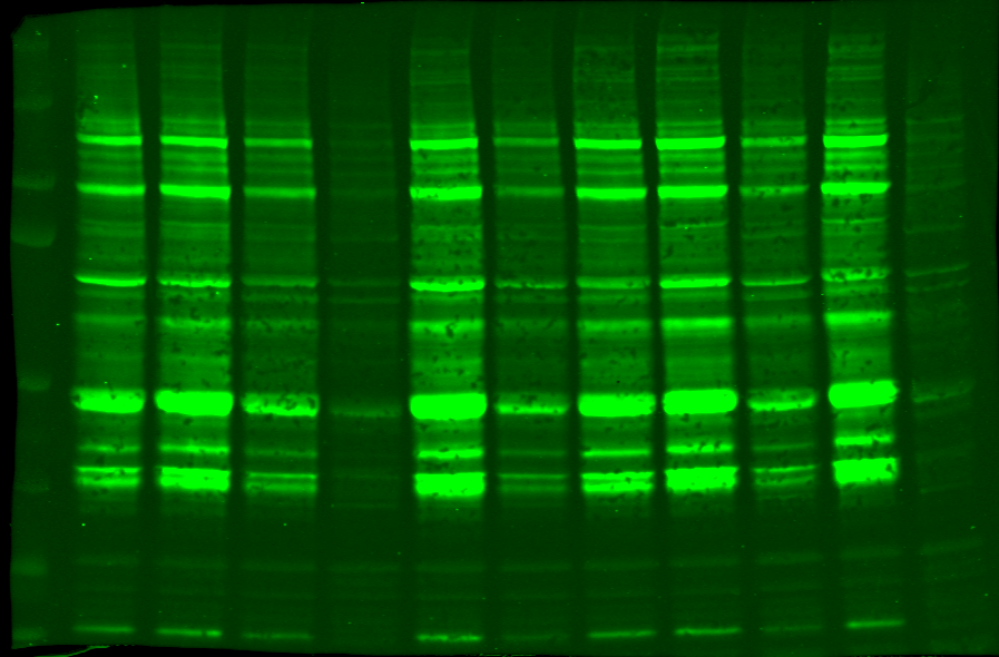

Supplement: Supplementary file 11 — Source data Fig. 4 [file 44320_2025_144_MOESM11_ESM.zip › Fig4/4B/20231128_1POK_new_no_MG_Ub_ub.png.tif]

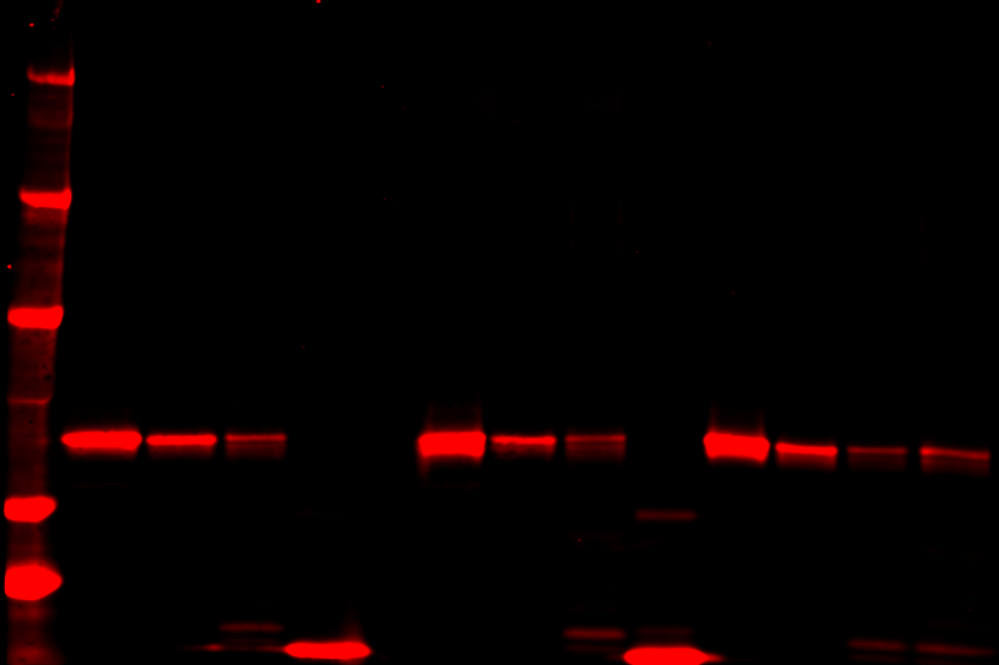

Supplement: Supplementary file 11 — Source data Fig. 4 [file 44320_2025_144_MOESM11_ESM.zip › Fig4/4C/1POK_YFP.tiff.tif]

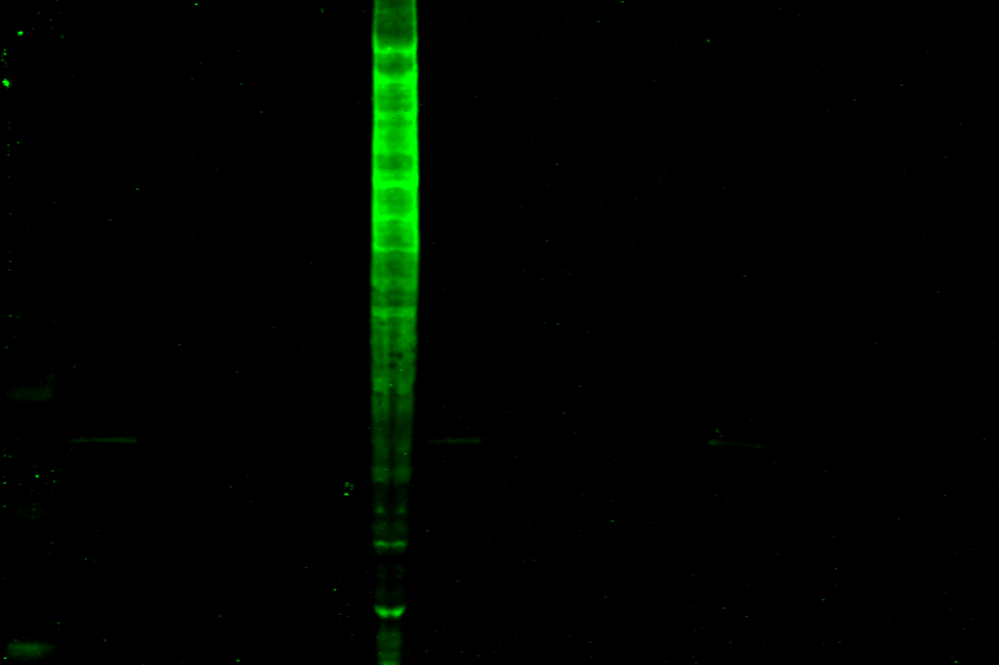

Supplement: Supplementary file 11 — Source data Fig. 4 [file 44320_2025_144_MOESM11_ESM.zip › Fig4/4C/1POK_ub.tiff.tif]

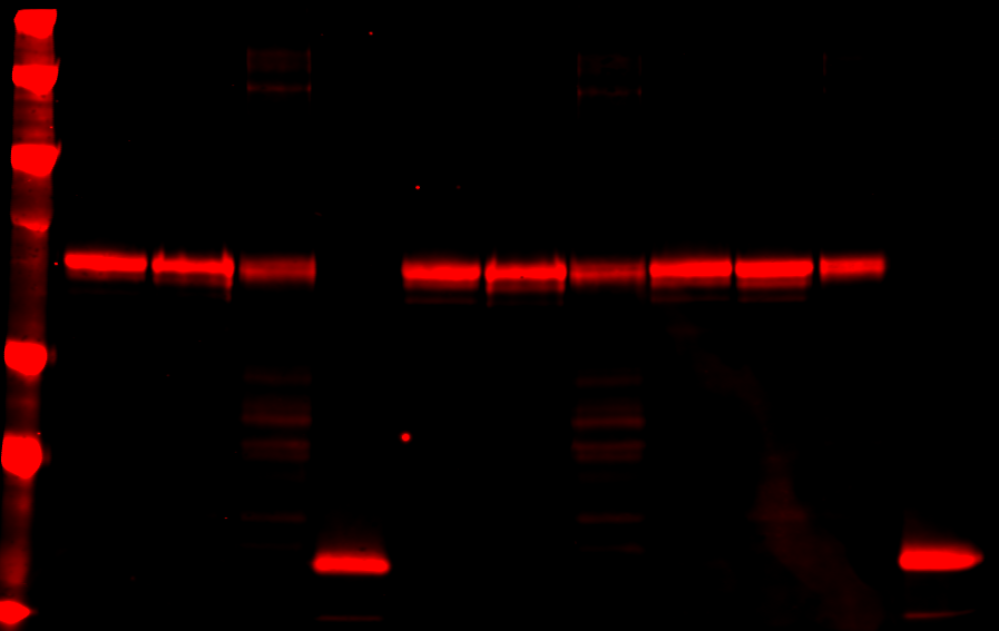

Supplement: Supplementary file 11 — Source data Fig. 4 [file 44320_2025_144_MOESM11_ESM.zip › Fig4/4D/1POK_lowcopy_yfp.tiff.tif]

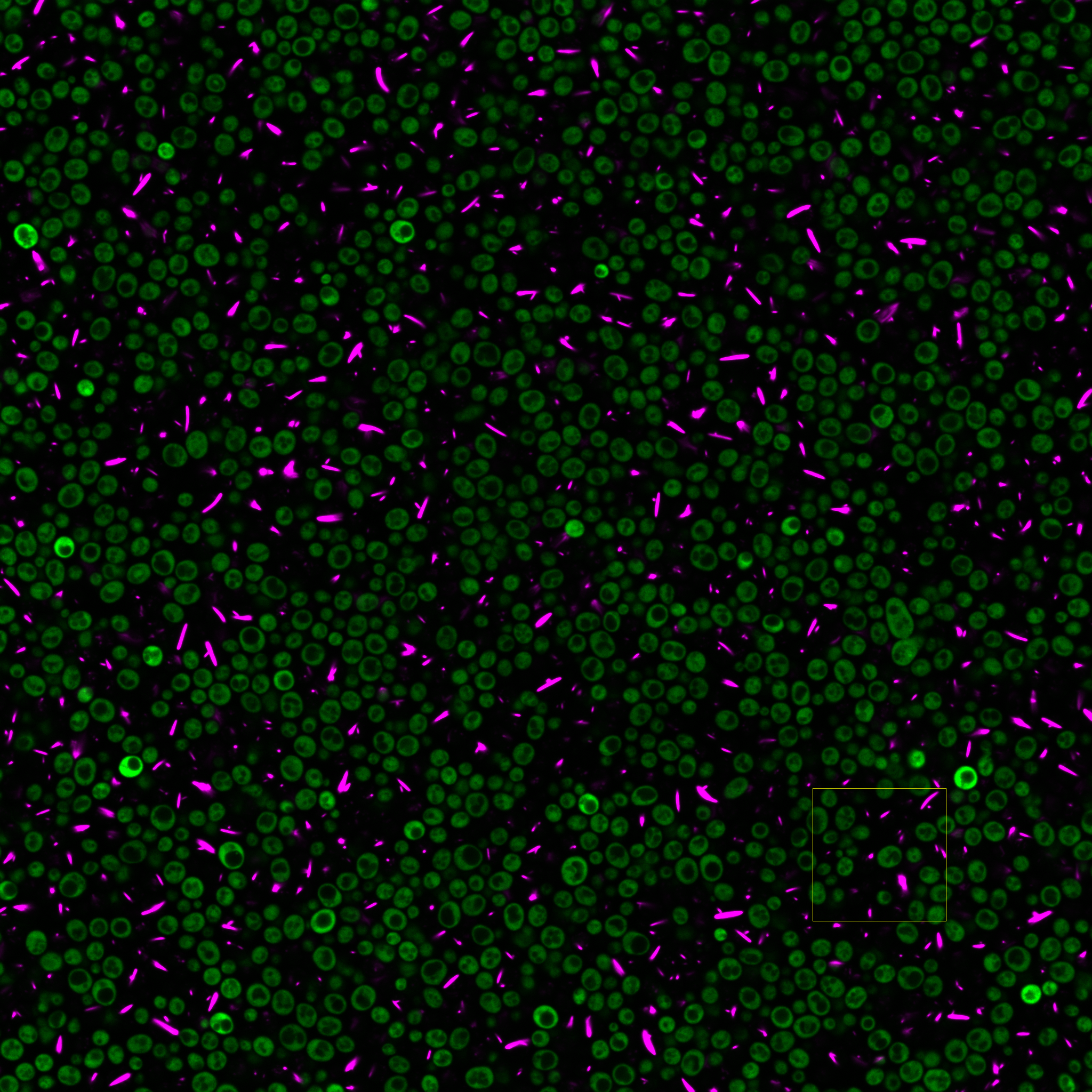

Supplement: Supplementary file 12 — Source data Fig. 5 [file 44320_2025_144_MOESM12_ESM.zip › Fig5/5B/WT-YFP_Fiber-Scarlet_comp.tif]

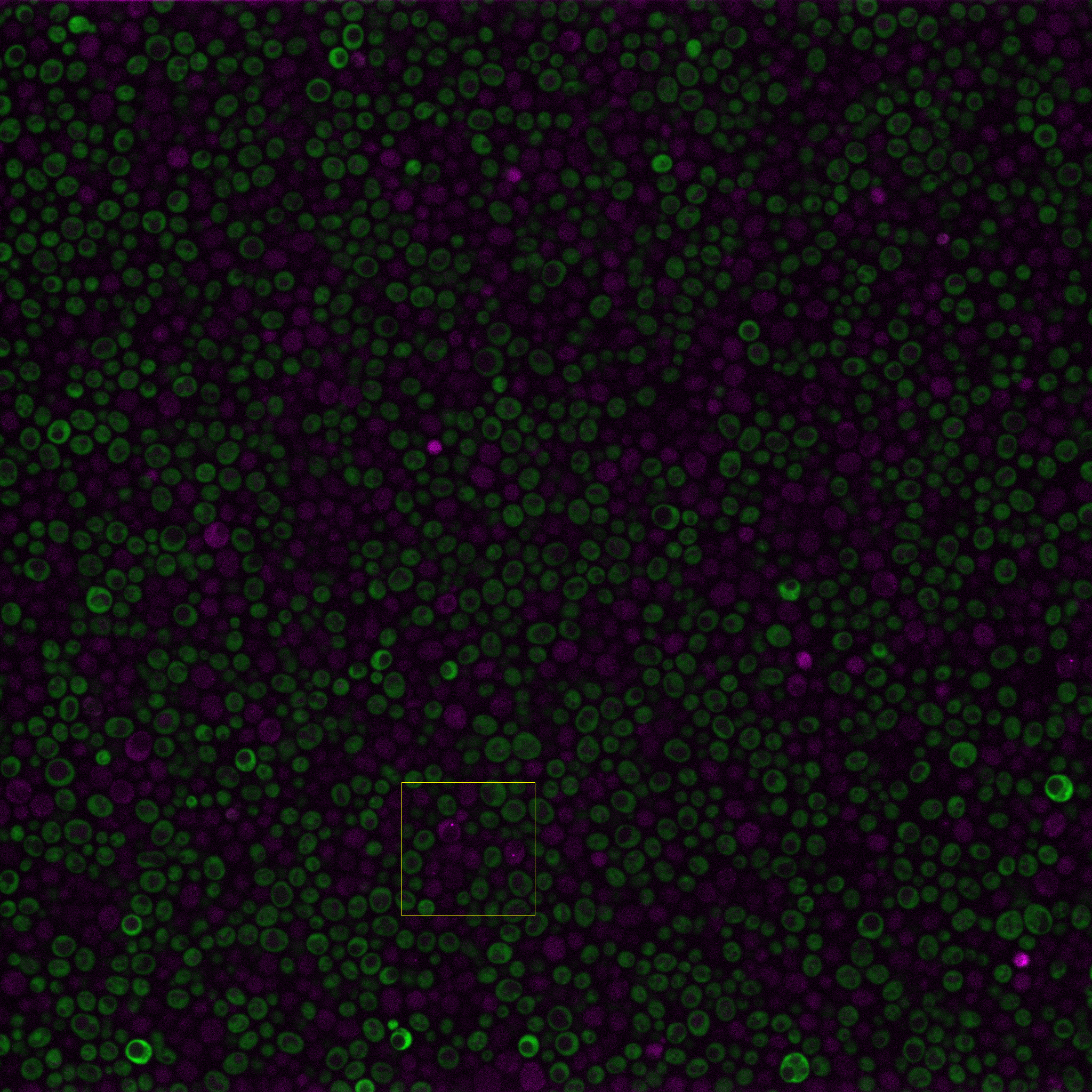

Supplement: Supplementary file 12 — Source data Fig. 5 [file 44320_2025_144_MOESM12_ESM.zip › Fig5/5B/WT-YFP_Misfolded-Scarlet_comp.tif]

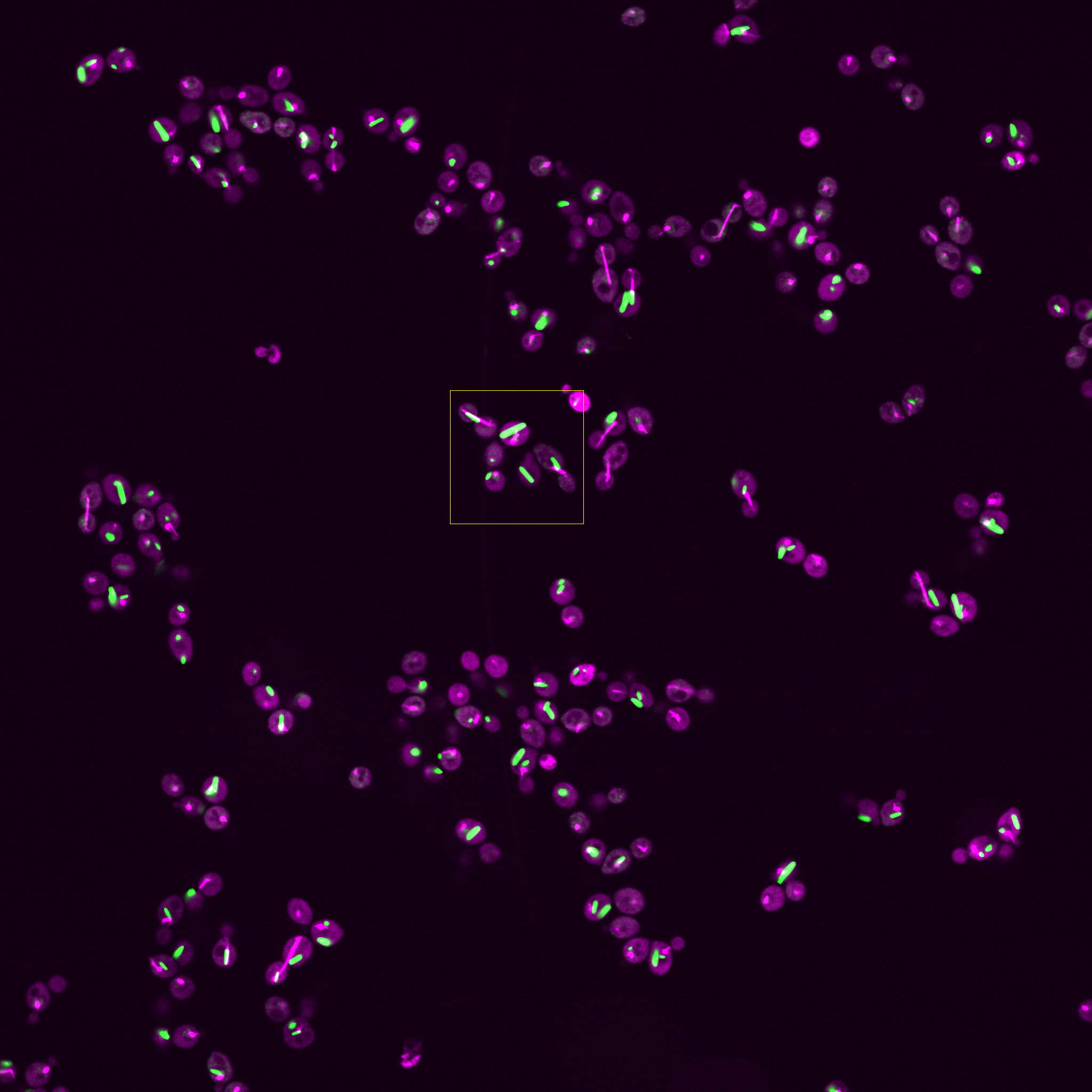

Supplement: Supplementary file 13 — Source data Fig. 6 [file 44320_2025_144_MOESM13_ESM.zip › Fig6/6F/2vyc-fiber-YFP_TUB1-mscarlet.tif]

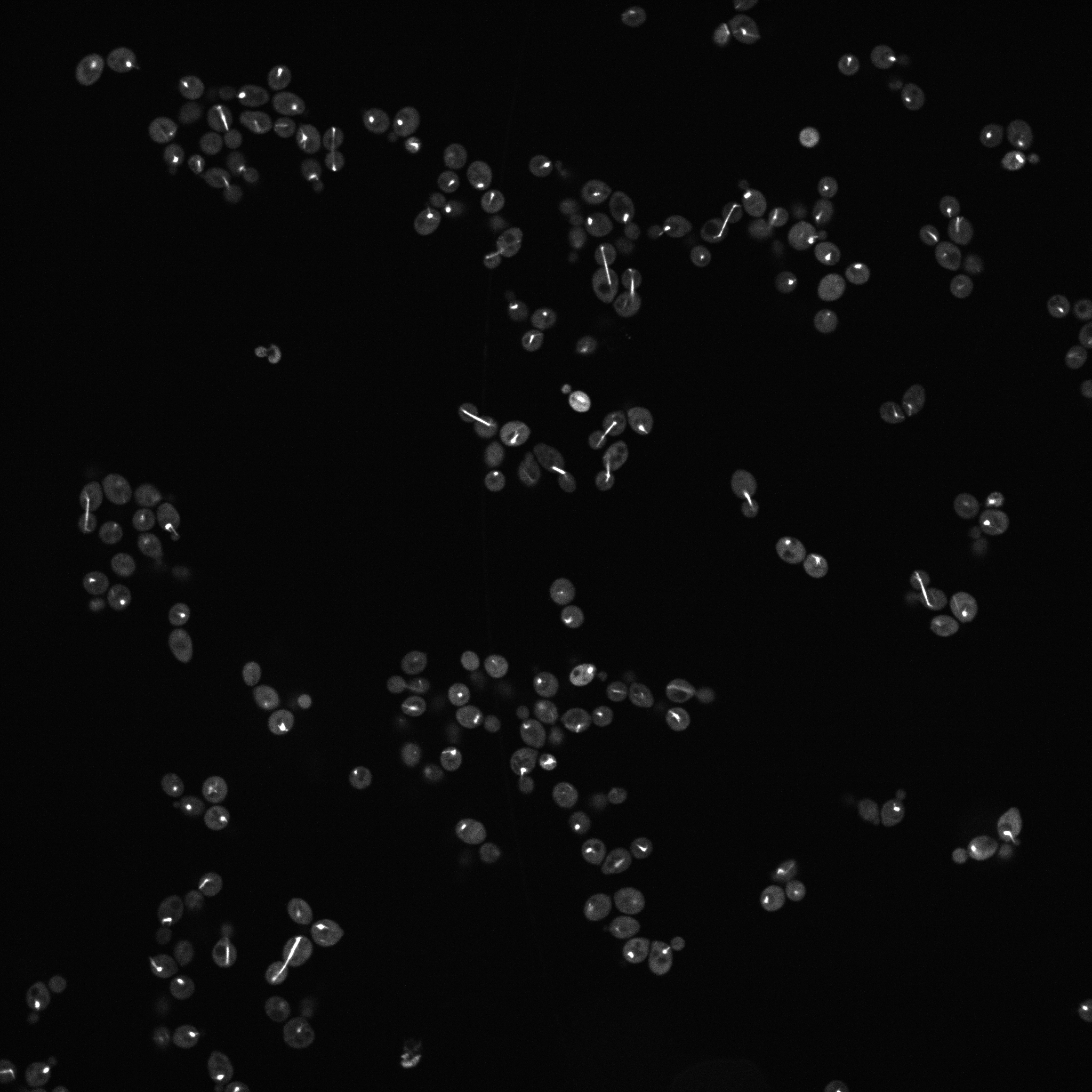

Supplement: Supplementary file 13 — Source data Fig. 6 [file 44320_2025_144_MOESM13_ESM.zip › Fig6/6F/2vyc_s2121_8align_cell12.tif]

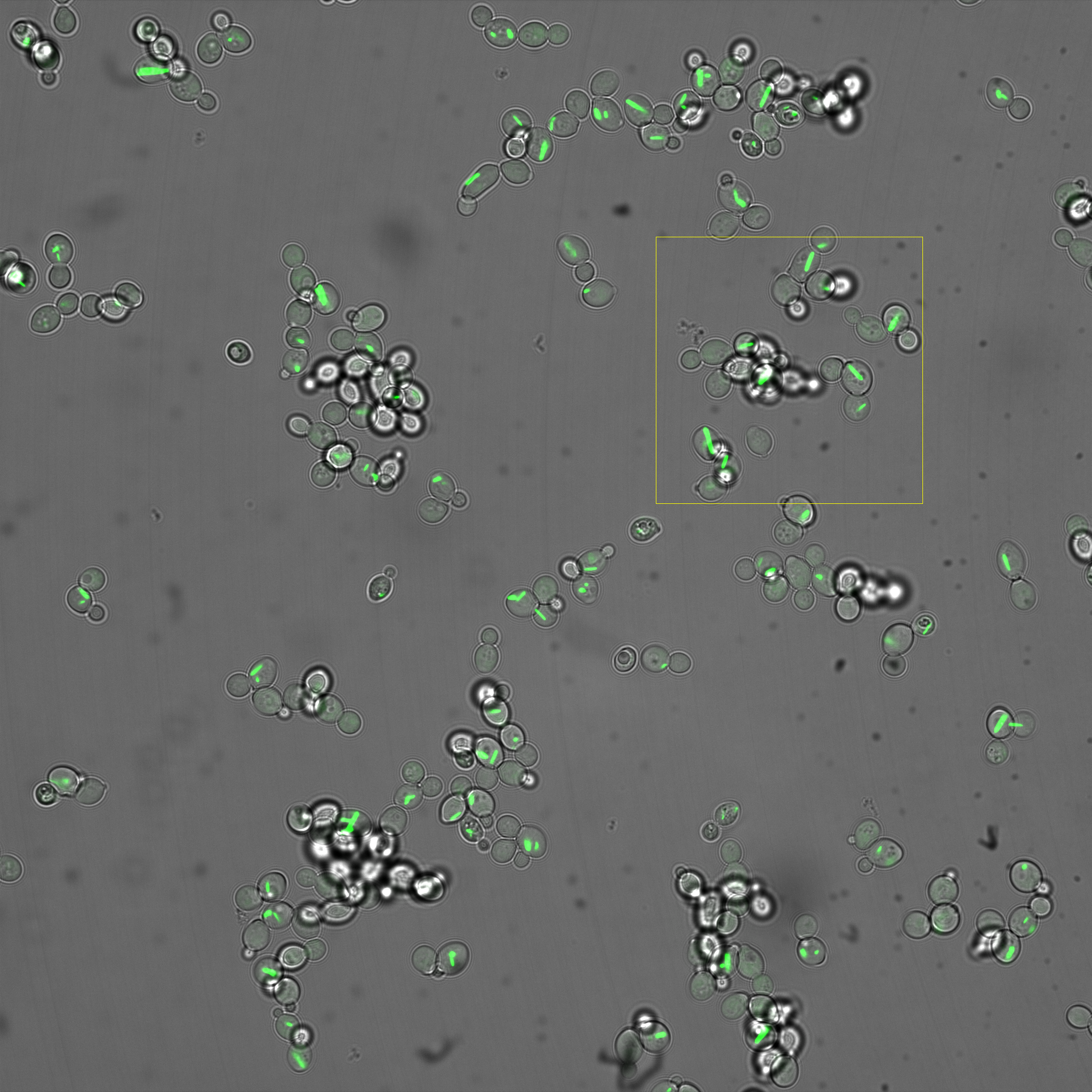

Supplement: Supplementary file 13 — Source data Fig. 6 [file 44320_2025_144_MOESM13_ESM.zip › Fig6/6E/2vyc_fiber.tif]
